# Hydroxylated TiO_2_-induced high-density Ni clusters for breaking the activity-selectivity trade-off of CO_2_ hydrogenation

**DOI:** 10.1038/s41467-024-52547-4

**Published:** 2024-09-27

**Authors:** Cong-Xiao Wang, Hao-Xin Liu, Hao Gu, Jin-Ying Li, Xiao-Meng Lai, Xin-Pu Fu, Wei-Wei Wang, Qiang Fu, Feng Ryan Wang, Chao Ma, Chun-Jiang Jia

**Affiliations:** 1 Key Laboratory for Colloid and Interface Chemistry, Key Laboratory of Special Aggregated Materials, School of Chemistry and Chemical Engineering, Shandong University, Jinan, 250100 China; 2 Department of Chemical Engineering, University College London, Roberts Building, Torrington Place, London, WC1E 7JE UK; 3 Hefei National Research Center for Physical Sciences at the Microscale, University of Science and Technology of China, Hefei, 230026 China; 4 School of Future Technology, University of Science and Technology of China, Hefei, 230026, China; 5 College of Materials Science and Engineering, Hunan University, Changsha, 410082 China

**Keywords:** Heterogeneous catalysis, Catalytic mechanisms, Catalyst synthesis

## Abstract

The reverse water gas shift reaction can be considered as a promising route to mitigate global warming by converting CO_2_ into syngas in a large scale, while it is still challenging for non-Cu-based catalysts to break the trade-off between activity and selectivity. Here, the relatively high loading of Ni species is highly dispersed on hydroxylated TiO_2_ through the strong Ni and −OH interactions, thereby inducing the formation of rich and stable Ni clusters (~1 nm) on anatase TiO_2_ during the reverse water gas shift reaction. This Ni cluster/TiO_2_ catalyst shows a simultaneous high CO_2_ conversion and high CO selectivity. Comprehensive characterizations and theoretical calculations demonstrate Ni cluster/TiO_2_ interfacial sites with strong CO_2_ activation capacity and weak CO adsorption are responsible for its unique catalytic performances. This work disentangles the activity-selectivity trade-off of the reverse water gas shift reaction, and emphasizes the importance of metal−OH interactions on surface.

## Abstract (web summary)

Here, the authors report a Ni(cluster)/TiO_2_ catalyst with strong interactions between Ni and −OH strong to promote CO_2_ activation with CO adsorption to avoid the activity-selectivity trade-off of the reverse water gas shift reaction.

## Introduction

As an available but inert source of carbon, greenhouse gas CO_2_ can be converted by hydrogen to form CO, which can be used as the feedstock for synthetic fuels^1–6^. Except for Cu-based catalysts, the selective hydrogenation of CO_2_ to CO, known as the reverse water gas shift (RWGS) reaction, is always accompanied by severe methanation, especially for the low-cost Ni-based catalysts with high C–O bond cleavage capacity^7–9^. Currently, methanation can be effectively inhibited through reported methods, including reducing metal loading^10–12^, forming metal carbide/phosphide^2,13^, applying the encapsulation layer over metal sites^14–16^, decreasing particle size^9^, and varying the support materials^17^. However, these methods decrease the density of surface active sites, leading to a significant activity trade-off for high CO selectivity. Therefore, it is important but challenging to develop a strategy to break such activity-selectivity trade-off over non-Cu-based catalysts^18–20^.

To enable a simultaneous high CO selectivity at high CO_2_ conversion, a catalyst must satisfy two key requirements^21^. On the one hand, sufficient active sites are required to activate and dissociate CO_2_^22–24^. On the other hand, active sites must possess relatively weak CO adsorption capacity, thus preventing the C–H bond formation and further hydrogenation to CH_4_^2,25^. It has been reported that compared to metal nanoparticles, supported metal clusters (SMCs) in high oxidation states have less adsorbing strength to CO, allowing a higher CO selectivity in CO_2_ hydrogenation^22,26^. However, the stability of SMCs is usually negatively correlated with their surface density under harsh CO_2_ hydrogenation conditions, leading to cluster sintering and rapid deactivation^9,27,28^.

As one typical reducible support, TiO_2_ with active surface oxygen has been widely used in supporting active metals to catalyze RWGS reaction^29,30^. Unfortunately, due to its limited ability to disperse metal species, metal loadings above 2 wt% usually trigger the aggregation of SMCs on TiO_2_ and, with selectivity, switch to methanation^31,32^. A feasible pathway to overcome the above challenges is to construct anchoring sites on the TiO_2_ surface that increase the binding strength to SMCs during the reaction. Recent reports suggest that surface hydroxyl (–OH) can be considered an effective anchoring site for dispersing active metals^33–36^. Even at room temperature, enriched surface –OH can effectively redisperse sintered metal particles into single atoms and clusters. Therefore, it can be expected that the sinter-resistant cluster catalyst can be designed by modifying the TiO_2_ surface with sufficient –OH, a method yet to be reported.

Herein, by converting the commercial anatase TiO_2_ into H_2_Ti_3_O_7_ tubes with abundant surface –OH groups, Ni species with relatively high loading (10 wt%) are dispersed as isolated atoms anchoring via the strong –OH-Ni binding force. Under the reducing atmosphere, accompanied by the removal of surface −OH and the reduction of Ni species, isolated Ni atoms are converted into stable clusters and a few TiO_*x*_-covered particles. In contrast, Ni species aggregate into large-size particles with poor activity on the reference commercial TiO_2_. Comprehensive characterizations and theoretical simulations confirm that due to the presence of Ni cluster/TiO_2_ interfaces with improved CO_2_ activation and weak CO adsorption, the activity-selectivity trade-off of the RWGS reaction is prevented, achieving simultaneous high CO_2_ conversion and high CO selectivity. The catalytic performance of this catalyst exceeds almost all reported values in RWGS, demonstrating that the hydroxylation of oxide supports can play an important role in developing high-performance supported catalysts.

## Results

### Formation and structure of the Ni-cluster/TiO_2_ catalyst

**Fig. 1:**
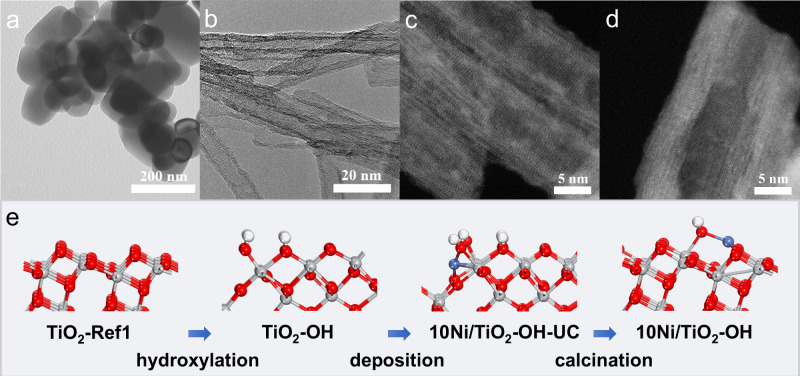
Structural information of catalyst. TEM images of **a** TiO_2_-Ref1 and **b** TiO_2_-OH samples. HAADF-STEM images of the **c** 10Ni/TiO_2_-OH-UC catalyst and **d** 10Ni/TiO_2_-OH catalyst. **e** Schematic illustration of the generation process for the 10Ni/TiO_2_-OH catalyst.

By using the commercial anatase TiO_2_ as a precursor (denoted by TiO_2_-Ref1, Fig. 1a), hydroxylated H_2_Ti_3_O_7_ was obtained as support for the Ni/TiO_2_ catalyst^37,38^. The detailed synthesis process and the corresponding catalyst structure evolution were shown as follows. Firstly, during a hydrothermal treatment under highly concentrated NaOH (10 mol/L) with subsequent HCl washing, a tube-shaped H_2_Ti_3_O_7_ phase (denoted by TiO_2_-OH, Fig. 1b and Supplementary Fig. 1) was prepared as the result of TiO_2_ reconstruction. Owing to the phase transformation, plenty of intrinsic –OH groups were identified on the surface of TiO_2_-OH, whereas very few –OH groups were present on the reference TiO_2_-Ref1 (Supplementary Fig. 2). Secondly, 10 wt% Ni was loaded on TiO_2_-OH by the deposition–precipitation method, and the obtained sample without air calcination was denoted by 10Ni/TiO_2_-OH-UC (Fig. 1c). The catalyst was subsequently calcinated in air at 600 °C. The hydroxylated H_2_Ti_3_O_7_ underwent dehydration, leading to a phase change back to anatase TiO_2_ (Supplementary Fig. 3). The attenuated total internal reflectance Fourier transform infrared spectroscopy (ATR-FTIR) test proved the maintenance of –OH groups on the surface even after calcination (Supplementary Fig. 4). Meanwhile, Ni was still well dispersed (denoted by 10Ni/TiO_2_-OH, Fig. 1d). The residual surface –OH on the as-prepared 10Ni/TiO_2_-OH catalyst improved the stability of isolated Ni atoms. The absence of Ni diffraction peaks in the X-ray diffraction (XRD) pattern for 10Ni/TiO_2_-OH implied the very small crystalline size of Ni species (Supplementary Fig. 3). The high dispersion of Ni over 10Ni/TiO_2_-OH was further confirmed by the atomic-resolution high-angle annular dark-field scanning transmission electron microscopy (HAADF-STEM) images and X-ray energy dispersive spectroscopy (EDS) elemental mapping results (Fig. 1d and Supplementary Fig. 5). The Fourier-transform extended X-ray absorption fine structure (EXAFS) results of 10Ni/TiO_2_-OH showed no Ni–Ni bond, demonstrating that Ni species were dispersed on the TiO_2_-OH support dominantly as single Ni atoms, though the presence of a small number of sub-nanometer Ni clusters could not be ruled out (Supplementary Fig. 6 and Supplementary Table 1). It was noteworthy that Ni single atoms on the catalyst surface were very sensitive to the radiation of high energy electron beam in TEM operating at 300 kV, and they would aggregate (<1 nm) with the increase of radiation time (Supplementary Fig. 7). Therefore, all the STEM images were collected within 10 s under small beam current. The schematic illustration of the generation process of 10Ni/TiO_2_-OH is depicted in Fig. 1e.

**Fig. 2:**
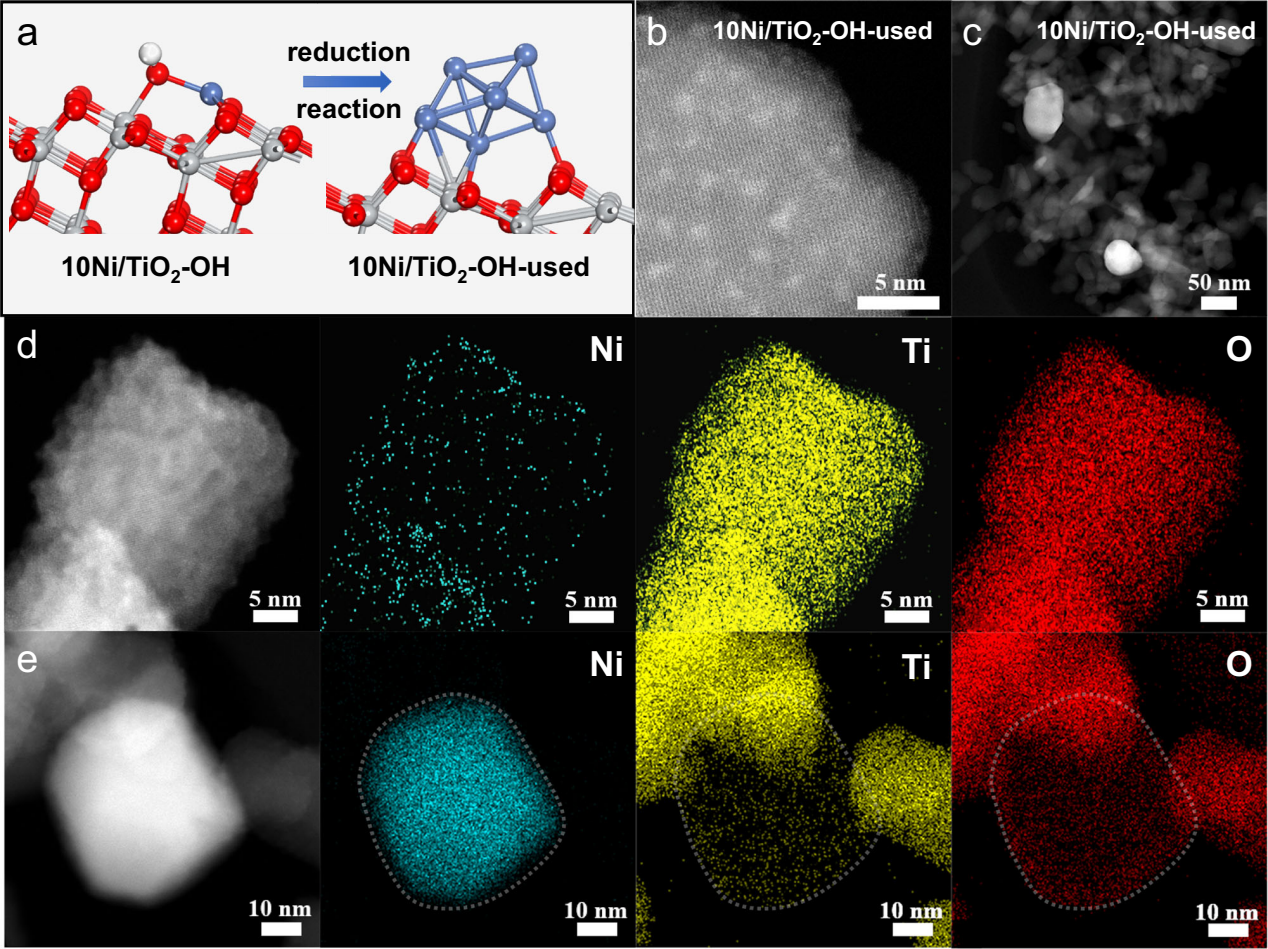
Structural information of catalyst. **a** Schematic illustration of the generation process for the used 10Ni/TiO_2_-OH catalyst from the as-prepared 10Ni/TiO_2_-OH catalyst. **b**, **c** HAADF-STEM images of the used 10Ni/TiO_2_-OH catalyst. The EDS-elemental mapping results for the **d** Ni cluster and **e** Ni particle of the used 10Ni/TiO_2_-OH catalysts.

Structure of the catalyst was further studied after the H_2_ pretreatment and CO_2_ hydrogenation (Fig. 2a). After the RWGS reaction (23%CO_2_/69%H_2_/8%N_2_) at 600 °C, isolated Ni atoms were converted into Ni clusters with the average size of ~1 nm (Fig. 2b and Supplementary Fig. 8) and a few large-sized Ni particles (Fig. 2c and Supplementary Fig. 9) on the sintered TiO_2_ support. The EDS-mapping results further indicated the good dispersion of Ni clusters, with Ni element appearing uniformly with Ti and O (Fig. 2d and Supplementary Fig. 10). Due to the strong metal-support interaction (SMSI)^39^, Ni particles were covered by a thin TiO_*x*_ overlayer (Fig. 2e and Supplementary Fig. 11), which has been widely reported in previous literatures^14,30,40^. The X-ray absorption near edge spectroscopy (XANES) of used 10Ni/TiO_2_-OH indicated the reduction of Ni species during the H_2_ pretreatment and the subsequent reaction process (Supplementary Fig. 6a). As illustrated by the EXAFS profiles (Supplementary Fig. 6b) and the 2-D contour plots wavelet transform (WT) results (Supplementary Fig. 6c, f), the obvious Ni–Ni contribution over used Ni/TiO_2_-OH suggested the aggregation of single Ni atoms, which was consistent with the XRD result (Supplementary Fig. 12).

In contrast, fresh and used Ni/TiO_2_-Ref1 catalysts exhibited distinctly different structures. Although the TiO_2_-Ref1 support morphology maintained well during the synthesis process, Ni particles with an average size of ~9.6 nm were formed on the catalyst surface after the air calcination at 600 °C (Supplementary Fig. 13). After the pretreatment and reaction, this catalyst further underwent severe sintering, and the average size of Ni particles increased to ~27.6 nm (Supplementary Fig. 14). Large-size Ni particles was further confirmed by the element mapping (Supplementary Fig. 15). Interestingly, based on ex-situ EELS-mapping results for 10Ni/TiO_2_-Ref1, no obvious TiO_*x*_ overlayer was found on Ni particles (Supplementary Fig. 16), which might be caused by the limited reducibility of TiO_2_-Ref1 (Supplementary Fig. 17). Based on above comprehensive experimental results, we provide a strategy for synthesizing Ni cluster catalysts by using hydroxylated TiO_2_ as support. Compared to commercial TiO_2_-Ref1 with poor ability to disperse Ni species, hydroxylated TiO_2_ with abundant –OH groups could effectively anchor Ni single atoms, thereby forming rich and durable Ni clusters even under harsh reaction conditions (600 °C and reducing atmosphere).

**Fig. 3:**
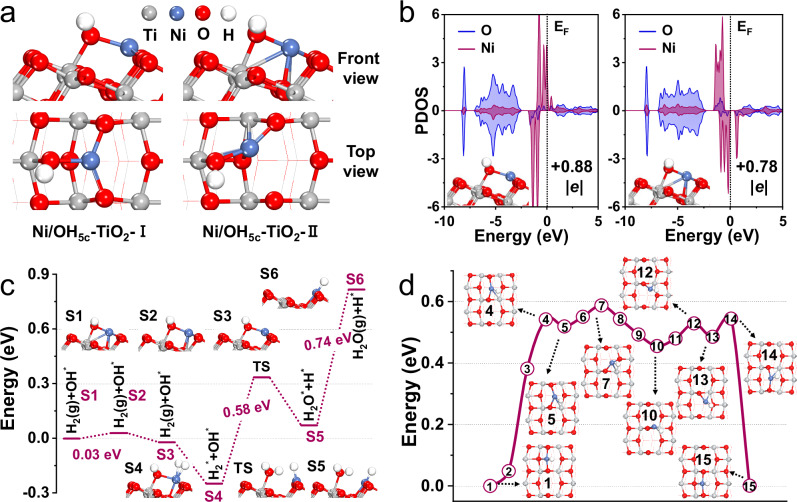
Structural evolution of catalyst. **a** Two most stable adsorption configurations of a single Ni atom on an OH_5c_/TiO_2_ surface (denoted as Ni/OH_5c_-TiO_2_-I and Ni/OH_5c_-TiO_2_-II, respectively), with the corresponding adsorption energies shown in Supplementary Table 2. **b** Projected electronic density of states (PDOS) of the single Ni atom and of the O atoms bonded with Ni in Ni/OH_5c_-TiO_2_-I (left) and Ni/OH_5c_-TiO_2_-II (right). The Fermi level was set to 0 eV. **c** Potential energy diagram for the removal of –OH group and the generation of the Ni–H species. Here, TS represents the transition state, and S1 and S3 correspond to Ni/OH_5c_-TiO_2_-II and Ni/OH_5c_-TiO_2_-I, respectively. **d** Potential energy diagram for the diffusion of the Ni-H species on the TiO_2_(101) surface.

In order to explore the mechanism of the effect of –OH groups on anchoring single Ni atoms under the air calcination, we carried out density functional theory (DFT) calculations. We adopted the anatase TiO_2_(101) surface to model the substrate. We noted that (101) was the most abundant surface of anatase TiO_2_^41^ that contained twofold coordinated O_2c_ atoms and fivefold coordinated Ti_5c_ atoms along the [010] direction (Supplementary Fig. 18). Supplementary Fig. 19 illustrated three types of hydroxylated TiO_2_(101) surfaces, in which the adsorption configurations of the Ni single atom were explored. The stable structures are shown in Fig. 3a, with the calculated adsorption energies listed in Supplementary Table 2. When single Ni atom adhered to an –OH group located at the fivefold coordinated Ti_5c_ atom (OH_5c_/TiO_2_, Supplementary Fig. 19a), the corresponding adsorption energies of Ni/OH_5c_-TiO_2_-I and Ni/OH_5c_-TiO_2_-II were higher than that on a bare anatase TiO_2_(101) surface (−5.83 eV, −5.81 eV vs. −2.89 eV). Notably, these values were also higher than the calculated cohesive energy of bulk Ni (−5.83 eV, −5.81 eV *vs*. −5.47 eV), meaning that the interaction between the Ni atom and the OH_5c_/TiO_2_ substrate was stronger than the interaction between Ni atoms in their bulk phase. Bader charge analysis (Supplementary Table 2) showed that the single Ni atom in Ni/OH_5c_-TiO_2_-I and Ni/OH_5c_-TiO_2_-II exhibited considerable positive charges (+0.88 |*e*| and +0.78 |*e*|), which enabled it to be stabilized via the electrostatic attraction with surrounding O atoms^42^. In addition, the projected electronic density of states of the Ni atom overlapped with that of the bonded O atoms (Fig. 3b), indicating that covalent interaction also existed between Ni and O. Hence, both electrostatic and covalent interactions within the Ni–O bonds contributed to the excellent stability of the adsorbed Ni atoms on the Ni/OH_5c_-TiO_2_-I and Ni/OH_5c_-TiO_2_-II models.

During the subsequent H_2_ pretreatment and the RWGS reaction, the potential mechanism of the transformation process from isolated Ni atoms to clusters was also explored by DFT simulations. Here, both Ni/OH_5c_-TiO_2_-I and Ni/OH_5c_-TiO_2_-II were considered as the initial states to investigate the subsequent structural changes of the Ni single atom. Under an H_2_ atmosphere, the −OH group in Ni/OH_5c_-TiO_2_-I could be easily removed by forming a water molecule with the H atom (Fig. 3c: S4 → TS → S5). Regarding Ni/OH_5c_-TiO_2_-II, although a direct removal of the –OH group (Supplementary Fig. 20: S2 → TS → S3) was a relatively hard process with an energy barrier of 1.06 eV, Ni/OH_5c_-TiO_2_-II could facilely transform to Ni/OH_5c_-TiO_2_-I with an energy barrier of only 0.03 eV (Fig. 3c: S1 → S2 →S3). It meant that the −OH removal in Ni/OH_5c_-TiO_2_-II could also occur easily, following the same pathway as that of Ni/OH_5c_-TiO_2_-I. Upon desorption of the water molecule (Fig. 3c: S5 → S6), a Ni–H species was left on the TiO_2_(101) surface. This Ni–H species could easily diffuse on the TiO_2_(101) surface, exhibiting an energy barrier of only 0.59 eV (Fig. 3d). As a comparison, the diffusion of an isolated Ni atom was also considered (Supplementary Fig. 21), and the corresponding energy barrier was calculated to be 1.03 eV. It could be seen that under reaction conditions, the H atom adsorbed on Ni could promote its diffusion, which was reminiscent of the effect of CO on the diffusion of Pt adatoms on Fe_3_O_4_(001)^43^. The removal of the –OH group and the promotion of the Ni diffusion under the H_2_ atmosphere well explained the agglomeration of Ni atoms to clusters observed after CO_2_ hydrogenation.

### Catalytic performance of the Ni/TiO_2_-OH catalysts with rich and stable Ni clusters

**Fig. 4:**
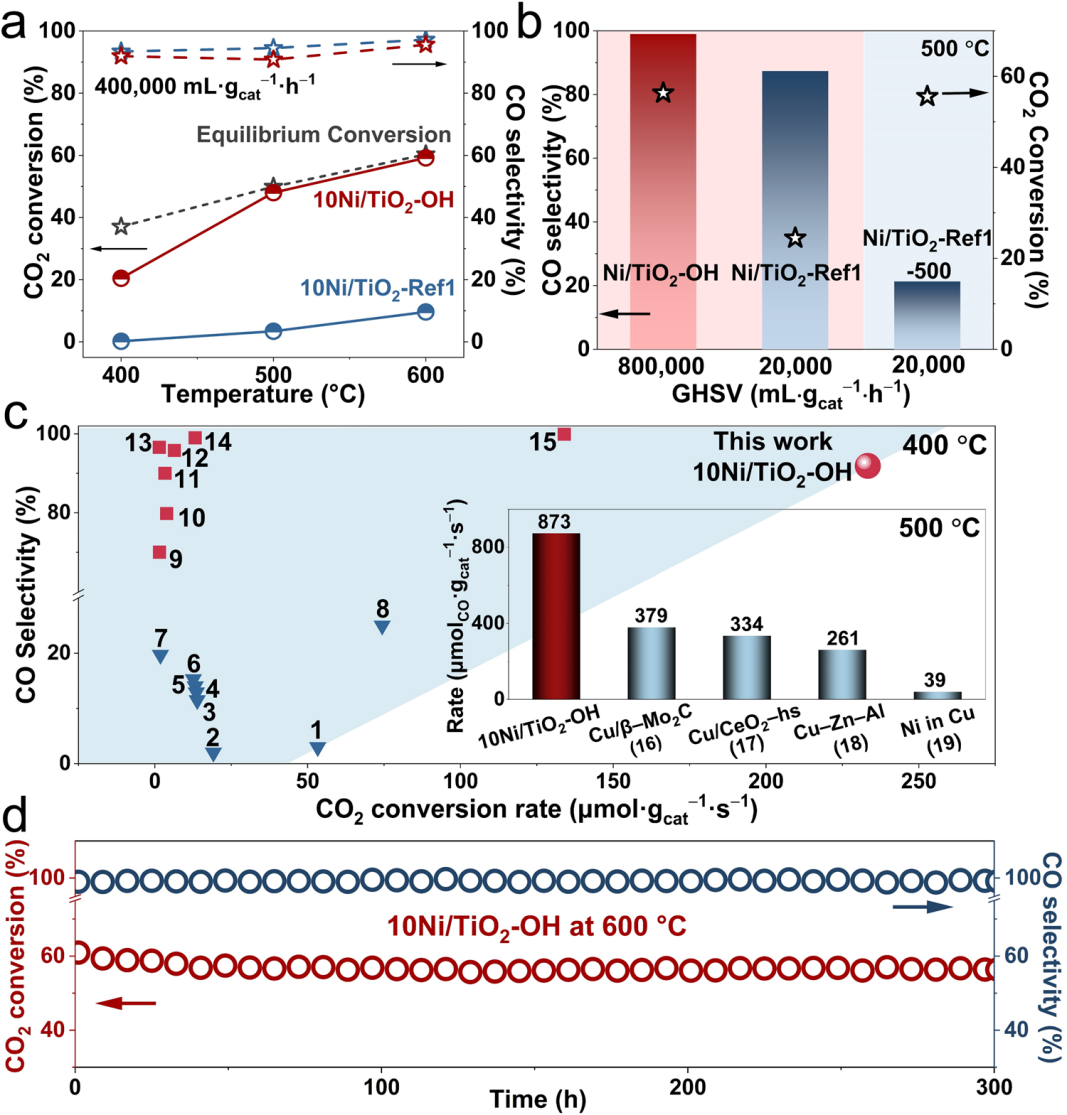
Catalytic performance for CO_2_ hydrogenation. **a** The CO_2_ conversion and CO selectivity for 10Ni/TiO_2_-OH and 10Ni/TiO_2_-Ref1 catalysts under the GHSV of 400,000 mL g_cat_^–1^ h^–1^. **b** Comparison of CO selectivity at 500 °C within relative comparable CO_2_ conversion rates for 10Ni/TiO_2_-OH, 10Ni/TiO_2_-Ref1 and 10Ni/TiO_2_-Ref1 after calcination at 500 °C and H_2_ pretreatment at 500 °C (10Ni/TiO_2_-Ref1-500). **c** CO_2_ conversion rates versus CO selectivity reported non-Cu based catalyst for CH_4_ production (blue triangles, Sel_CO_ < 50%) and CO production (red squares, Sel_CO_ > 50%), and this work (red circle) at 400 °C. Inset: comparison of CO yield rates over 10Ni/TiO_2_-OH catalysts and Cu-based catalysts at 500 °C. **d** The long-term stability of the 10Ni/TiO_2_-OH catalyst tested for 300 h at 600 °C (GHSV = 800,000 mL g_cat_^−1^ h^−1^).

It has been reported that compared to large-size Ni particles, Ni clusters with poor electronic transfer to CO molecules can promote the desorption of carbonyl (CO*) species to form gaseous CO molecules^9^. Therefore, it can be expected that the obtained 10Ni/TiO_2_-OH catalyst with abundant Ni clusters could exhibit high catalytic performance to catalyze the RWGS reaction. As shown in Fig. 4a, 10Ni/TiO_2_-OH had high CO_2_ conversion, which was comparable to the thermodynamic equilibrium limitation at 500 °C and 600 °C. However, due to the aggregated Ni particles, the CO_2_ conversion of 10Ni/TiO_2_-Ref1 was less than 10% even at 600 °C. Meanwhile, 10Ni/TiO_2_-OH showed excellent CO selectivity, maintaining above 90% at all reaction temperatures, in clear contrast to the generally observed near 100% selectivity towards methane which is generally observed on nickel-based catalysts. In order to further compare the product selectivity of 10Ni/TiO_2_-OH and 10Ni/TiO_2_-Ref1 with distinctly different Ni structures, additional activity tests were performed. Through controlling GHSV, we tried to compare the product selectivity of 10Ni/TiO_2_-OH and 10Ni/TiO_2_-Ref1 under a similar CO_2_ conversion. As shown in Fig. 4b, even when the GHSV was increased to 800,000 mL g_cat_^−1^ h^−1^, the CO_2_ conversion of 10Ni/TiO_2_-OH was still much higher than that of 10Ni/TiO_2_-Ref1 with a much lower GHSV of 20,000 mL g_cat_^−1^ h^−1^. Under such test conditions, the CO selectivity of 10Ni/TiO_2_-Ref1 was slightly lower but still over 85% compared to that of 10Ni/TiO_2_-OH, over 98%, which seemed to conflict with the previously reported results that large-size metal particles led to the high CH_4_ selectivity^9^. To resolve this confusion, by reducing the air calcination temperature and the subsequent pretreatment temperature of 10Ni/TiO_2_-Ref1 from 600 °C to 500 °C, we further synthesized another reference catalyst with relative smaller Ni particles than 10Ni/TiO_2_-Ref1 and referred to as 10Ni/TiO_2_-Ref1-500, in which Ni particle size was ~12.4 nm (Supplementary Fig. 22). As the particle size decreased, the catalytic activity was improved compared to 10Ni/TiO_2_-Ref1, but it was still lower than that of 10Ni/TiO_2_-OH with abundant Ni clusters (Supplementary Fig. 23). Importantly, at similar CO_2_ conversion rates, the CO selectivity of 10Ni/TiO_2_-OH (98.9%) was much higher than that of 10Ni/TiO_2_-Ref1-500 (21.3%), indicating the selectivity difference between clusters and particles in catalyzing CO_2_ hydrogenation (Fig. 4b). Based on above results, the relationship between the Ni size on TiO_2_ and the product selectivity in atmospheric CO_2_ hydrogenation could be clearly revealed. On the one hand, compared to highly dispersed Ni clusters, Ni particles induced the formation of CH_4_. In addition, when the average size of Ni particle was larger than 27 nm, it was meaningless to discuss the product selectivity due to the very poor catalytic activity.

As reported, extensive efforts have been made to optimize the CO selectivity of non-Cu catalysts by regulating the catalyst structure^2,10–16^. However, due to the presence of an activity-selectivity trade-off, it was very challenging to achieve the combination of high activity and high selectivity (non-Cu-based catalysts ordered 1–15^13,23,44–48^ in Fig. 4c and Supplementary Table 3). Obviously, 10Ni/TiO_2_-OH successfully broke the trade-off between activity and selectivity with a high CO selectivity of 91.9% while maintaining an excellent reaction rate of 214.4 μmol g_cat_^−1^ s^−1^ at 400 °C. Furthermore, compared to Cu-based catalysts, which were regarded as prevailing catalysts for catalyzing the RWGS reaction, 10Ni/TiO_2_-OH with an excellent reaction rate of 873.0 μmol_CO_ g_cat_^−1^ s^−1^ and a high CO selectivity of 98.0% at 500 °C was also highly advantageous (Cu-based catalysts ordered 16–19^6,49,50^ in inset of Fig. 4c and Supplementary Table 3). The apparent activation energy (*E*_*a*_) and the corresponding CO_2_ conversion and CO selectivity for 10Ni/TiO_2_-OH were shown in Supplementary Fig. 24. Besides, the catalytic performance of 10Ni/TiO_2_-OH was evaluated in different reaction atmospheres with various H_2_ to CO_2_ ratios. With the increase of the ratio of H_2_ to CO_2_, CO_2_ conversion increased slightly (Supplementary Fig. 25a), suggesting this catalyst possessed a high catalytic activity over a wide input reaction gas composition. Moreover, it was found that the CO selectivity maintained over 90% even when the H_2_ to CO_2_ ratio reached 4:1 (Supplementary Fig. 25b), which indicated that the methanation was indeed inhibited by this catalyst. Due to the harsh reaction conditions of the high-temperature RWGS reaction, reported supported catalysts are always severely deactivated by the catalyst sintering^51,52^. In this work, the long-term stability of 10Ni/TiO_2_-OH was evaluated. As shown in Fig. 4d, under very harsh reaction conditions (600 °C and GHSV = 800,000 mL g_cat_^−1^ h^−1^), 10Ni/TiO_2_-OH remained stable with the CO_2_ conversion more than ~55% and showed almost complete CO selectivity. Besides, 10Ni/TiO_2_-OH also suggested a high start-up cool-down stability (Supplementary Fig. 26a). In contrast, under relatively milder reaction conditions, 10Ni/TiO_2_-Ref1 lost almost all of its activity in only 2.5 h (Supplementary Fig. 26b), which was accompanied by the significant increase in particle size (Supplementary Fig. 27), further demonstrated the importance of suppressing the Ni sintering for reaction activity. Based on the above results, 10Ni/TiO_2_-OH with rich Ni clusters efficiently broke the activity-selectivity trade-off of the RWGS reaction and achieved a combination of high activity, high CO selectivity and excellent stability. To the best of our knowledge, the catalytic performance of 10Ni/TiO_2_-OH was unmatched by almost all other reported catalysts employed for the atmospheric high-temperature RWGS reaction.

The structure of the catalyst after 300 h of steady-state reaction at 600 °C was explored by HAADF-STEM. As shown in Supplementary Fig. 28, Ni clusters were still anchored on the catalyst surface. This result again emphasized the efficiency of –OH groups on the TiO_2_-OH support for constructing active Ni structure.

### Strong interaction between the Ni cluster and TiO_2_-OH

**Fig. 5:**
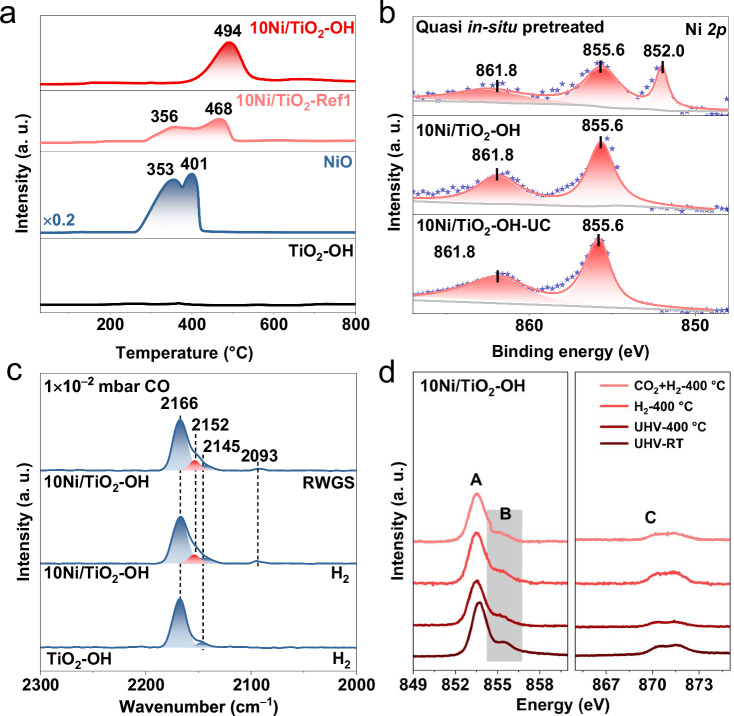
Interfacial structure of Ni/TiO_2_ catalyst. **a** H_2_-TPR profiles of the NiO, 10Ni/TiO_2_-Ref1 and 10Ni/TiO_2_-OH samples. **b** XPS spectra and corresponding fitting curves of Ni *2p* in the 10Ni/TiO_2_-OH-UC and 10Ni/TiO_2_-OH catalysts. Quasi in situ XPS spectra and corresponding fitting curves of Ni *2p* in 10Ni/TiO_2_-OH catalyst after RWGS reaction at 600 °C for 3 h. **c** In situ infrared spectra recorded after exposing the TiO_2_-OH after H_2_ pretreatment and 10Ni/TiO_2_-OH catalysts after H_2_ pretreatment and RWGS reaction to CO at 130 K with 1 × 10^−^^2^ mbar. **d** The in-situ Ni L-edge NAP-NEXAFS profile of the 10Ni/TiO_2_-OH catalyst.

It has been established that catalyst structures and catalytic performances are largely determined by the MSI. Therefore, it is essential to reveal the interaction between Ni species and the TiO_2_-OH support for 10Ni/TiO_2_-OH, thereby understanding the stable interfacial structure and the molecule activation behavior. Firstly, we used the temperature-programmed hydrogen reduction (H_2_-TPR) technique to investigate the redox properties of the 10Ni/TiO_2_-OH catalyst. As shown in the H_2_-TPR profile (Fig. 5a), pure NiO exhibited two reduction peaks centered at 352 °C and 401 °C, which could be attributed to the cascading reduction of Ni^2+^ to Ni^+^ and then to Ni^0^. 10Ni/TiO_2_-Ref1 showed similar reduction features to that of pure NiO, suggesting a very weak interaction between Ni species and TiO_2_-Ref1. Compared to pure NiO, the increased reduction temperatures for 10Ni/TiO_2_-Ref1 might be caused by the size difference of NiO. The reduction peak of the 10Ni/TiO_2_-OH sample was centered at 494 °C, much higher than that of the above two samples, which indicated a higher energy barrier to break the Ni-O bond. By quantifying the consumed H_2_ based on H_2_-TPR results (Supplementary Fig. 29), it could be found that the reduction peaks of 10Ni/TiO_2_-Ref1 were mainly from the NiO reduction to Ni since the actual hydrogen consumption was close to the theoretical value. This again confirmed the weak MSI between Ni species and TiO_2_-Ref1. However, the actual H_2_ consumption of 10Ni/TiO_2_-OH was much higher than the theoretical H_2_ consumption, suggesting that partial surface oxygen atoms of TiO_2_-OH support were reduced to generate TiO_*x*_ structure. According to previous literature, the formation of TiO_*x*_ could cover the large-size metal particles^53,54^, so large-size Ni particles on 10Ni/TiO_2_-OH were covered by TiO_*x*_, while the same phenomenon was not found on 10Ni/TiO_2_-Ref1. The above analysis of H_2_-TPR results clearly revealed that TiO_2_-OH with abundant –OH groups could induce the formation of the enhanced interaction between Ni and TiO_2_-OH, promoting the sintering-resisting capacity of Ni clusters on the TiO_2_ surface. Actually, in addition to anchoring Ni species, such a strong interaction stabilized the TiO_2_. Unlike pure TiO_2_-OH supports seriously sintered after the air calcination at 500 °C (Supplementary Fig. 30a, b), TiO_2_ in 10Ni/TiO_2_-OH maintained the tube-shape morphology well (Supplementary Fig. 30c, d).

The strong interaction between Ni and TiO_2_ in 10Ni/TiO_2_-OH was further verified by the following quasi in situ XPS, in situ infrared spectra (IR), and in situ near ambient pressure (NAP) near edge X-ray absorption fine structure (NEXAFS). From the Ni *2p* XPS spectra (Fig. 5b), the Ni *2p*_*1/2*_ binding energy (B.E.) of as-prepared 10Ni/TiO_2_-OH was at ~855.6 eV, which was attributed to the presence of Ni^2+^^2^. After the RWGS reaction, besides the Ni^2+^ species at ~855.6 eV, the Ni^0^ species at ~852.0 eV also appeared^22^, which suggested the reduction of Ni^2+^ under the reducing atmosphere. The Ni^2+^/(Ni^0^ + Ni^2+^) ratio was calculated to be ~66%, indicating that even after the treatment by the reaction gas with highly concentrated H_2_ of 69%, a majority of Ni^2+^ species still existed on the catalyst surface. The presence of abundant Ni^2+^ species represented the electronic metal-support interaction (EMSI) between Ni species and TiO_2_, which prevented Ni species from agglomerating on the TiO_2_ support surface caused by the easy reduction. Because the Ni species in 10Ni/TiO_2_-OH with excellent CO selectivity featured a weak adsorption capacity for CO molecules, it was difficult to use CO molecules as probes to characterize the state of Ni species by in situ DRIFTS at room temperature. In general, the decrease in temperature could improve the adsorption strength of metal species for CO molecules, so as to more clearly characterize the state of metal species^55^. Therefore, in situ IR spectra of CO adsorption were collected at a low testing temperature of 130 K with a pressure of 1 × 10^−2^ mbar (Fig. 5c). Pristine TiO_2_-OH pretreated with H_2_ exhibited a main peak at 2168 cm^–1^ with a shoulder peak at 2147 cm^–1^, ascribed to the adsorption of CO on distinct exposed facets of TiO_2_^56^. By contrast, there were two extra peaks in the Ni/TiO_2_-OH catalyst. One at 2152 cm^−1^ was assigned to CO linearly adsorbed on Ni^2+^ sites^57,58^. In addition, the signal at 2090 cm^−1^ likely corresponded to sub-carbonyl Ni(CO)_*x*_ (*x* = 2 or 3) species, which featured a highly disordered structure and were attributed to amorphous Ni sites^59,60^. Similar results were observed as the CO partial pressure was reduced to 1 × 10^−7^ mbar (Supplementary Fig. 31). The dominant Ni^2+^-CO signal confirmed the presence of rich Ni^2+^, which again presented the strong EMSI between Ni and TiO_2_. Moreover, the sub-carbonyl species were weakly adsorbed, which was favored by CO removal, thereby facilitating the high CO selectivity of the RWGS reaction. Ni L-edge NAP-NEXAFS was further measured to detect the surface Ni species under various atmospheres. Auger electron yield (AEY) for Ni L_3_ (853 eV to 856 eV) and L_2_ (870 eV to 872 eV) was observed (Fig. 5d), which were related to *2p*_3/2_ to *3d* and *2p*_1/2_ to *3d* transition, respectively. For 10Ni/TiO_2_-OH, the relatively stable Ni L edge signals suggested the high stability of Ni^2+^ even under H_2_ and RWGS reaction flows at 400 °C, which further indicated the EMSI between Ni and the support. In comparison, 10Ni/TiO_2_-Ref1 showed a significant change in intensity at 855.5 eV under the H_2_ atmosphere at 300 °C, demonstrating that Ni^2+^ was easily reduced to Ni^0^ (Supplementary Fig. 32). The surface structure of the TiO_2_ support over 10Ni/TiO_2_-OH was also explored by XPS and NAP-NEXAFS. The Ti *2p* XPS spectra exhibited that even after the treatment by the RWGS reaction flow at 600 °C, only Ti^4+^ species but no obvious Ti^3+^ signal could be found (Supplementary Fig. 33a). The in situ Ti L-edge NAP-NEXAFS profile of 10Ni/TiO_2_-OH also did not detect the formation of Ti^3+^ under H_2_ and reaction flows (Supplementary Fig. 33b). Based on above results, we speculated that even though the MSI between Ni and TiO_2_ facilitated the partial reduction of TiO_2_, the limited concentration of surface Ti^3+^ sites were below the detection line of XPS and in situ NEXAFS techniques. The created Ti^3+^ might be moved into the bulk phase of anatase TiO_2_^61^.

### The proposed reaction mechanism of CO_2_ hydrogenation catalyzed by Ni/TiO_2_-OH

**Fig. 6:**
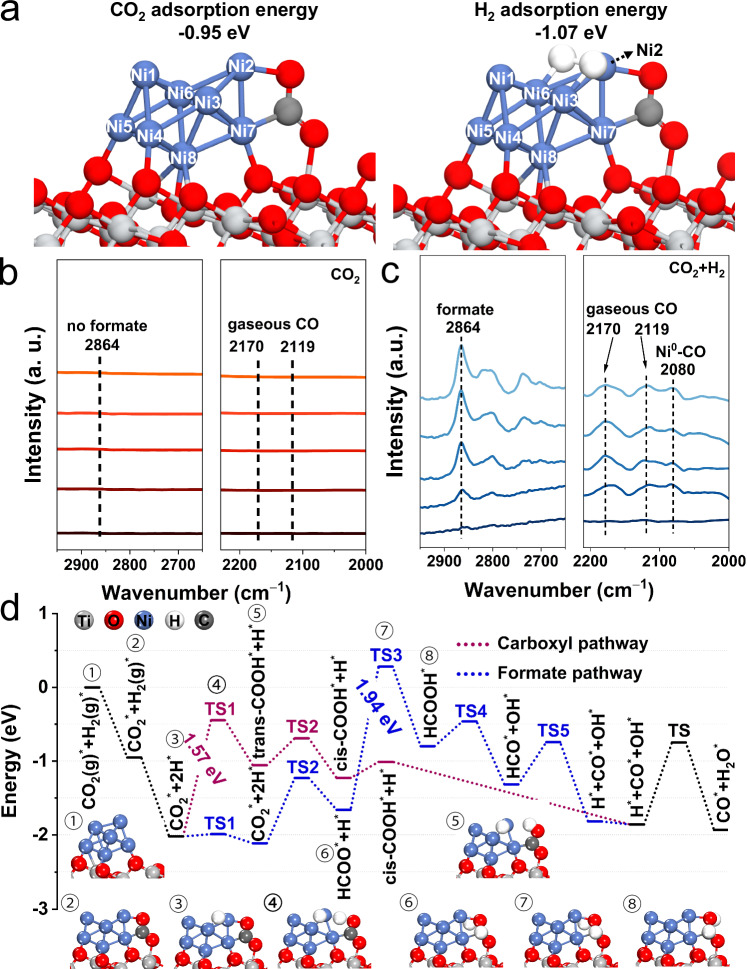
Theoretical and experimental results for the reaction mechanism. **a** CO_2_ adsorption on Ni_8_/TiO_2_ system and H_2_ adsorption on CO_2_-Ni_8_/TiO_2_ system. **b**, **c** In situ diffused reflectance infrared Fourier transform spectroscopy (DRIFTS) spectra of 10Ni/TiO_2_-OH catalyst during CO_2_ treatment and reaction conditions (CO_2_ + H_2_) at 300 °C, respectively. **d** Potential energy diagram for the RWGS reaction on Ni_8_/TiO_2_(101) via carboxyl and formate pathways. The illustration showed the structures of some intermediate and transition states. The complete reaction pathways are shown in Supplementary Figs. 38 (carboxyl) and 39 (formate). “TS” represents the transition state.

To identify the potential reaction mechanism of CO_2_ hydrogenation to CO catalyzed by this highly performed 10Ni/TiO_2_-OH catalyst, we first evaluated the CO_2_ adsorption ability through the temperature-programmed desorption of CO_2_ (CO_2_-TPD) (Supplementary Fig. 34). Compared to 10Ni/TiO_2_-Ref1 with weak capacity to adsorb CO_2_, two strong CO_2_ desorption peaks appeared over 10Ni/TiO_2_-OH. By comparing the result of Ar-TPD, the first feature at low temperatures could be ascribed to chemisorbed CO_2_ molecules, and the latter one was related to the decomposition of surface carbides. The strong CO_2_ desorption signal indicated that compared to 10Ni/TiO_2_-Ref1, rich Ni cluster-TiO_2_ interfaces might serve as crucial sites for absorbing CO_2_. Subsequently, DFT calculations were performed to further understand the adsorption and activation of CO_2_ on this Ni cluster catalyst through a catalyst model of Ni_8_/TiO_2_ surface (Ni_8_/TiO_2_ model seen Supplementary Fig. 35). It should be noted that because no distinct Ti^3+^ signal was detected on the catalyst surface, we did not consider the presence of oxygen vacancies in this constructed catalyst model. As shown in Fig. 6a, CO_2_ was easily adsorbed at the Ni cluster-TiO_2_ interface with an adsorption energy of −0.95 eV, and the Ni_8_ cluster became ship-shaped. Meanwhile, H_2_ was spontaneously dissociated into two H* on the Ni cluster. This result declared that the Ni cluster/TiO_2_ interface, which was ultra-stable in the RWGS reaction, could be considered as a significant active structure to activate reactant molecules.

Moreover, we also explored the CO_2_ hydrogenation mechanism of 10Ni/TiO_2_-OH. The mechanisms of the RWGS reaction have been categorized into two types: redox mechanism and associative mechanism. The dissociation experiment of CO_2_ was conducted to investigate the reaction pathway. After the H_2_/Ar pretreatment at 600 °C, the CO_2_/Ar mix gas was injected into the reactor after the sample was cooled to room temperature. However, there was no generation of CO and consumption of CO_2_ during the subsequent warming process, which indicated that CO might not be produced by the direct dissociation of CO_2_ on the 10Ni/TiO_2_-OH catalyst (Supplementary Fig. 36a). In contrast, the temperature-programmed surface reaction (TPSR) results showed that the CO_2_ signal gradually decreased start from 300 °C, accompanied by the increase of CO signal, demonstrating that the dissociation of CO_2_ into CO required the assistance of H_2_ (Supplementary Fig. 36b). By combining the results of the CO_2_ dissociation experiment and TPSR, it could be inferred that CO_2_ activation likely occurred through an associative intermediate pathway rather than the redox mechanism. Similar results were revealed by in situ diffuse reflectance infrared Fourier transform spectroscopy (DRIFTS). After CO_2_ injection, only carbonate species appeared, without signal of formate and CO signal, which further proved that it was difficult for CO_2_ to be directly dissociated to CO (Fig. 6b and Supplementary Fig. 37a). Subsequently, H_2_ flow was introduced to the 10Ni/TiO_2_-OH catalyst, a new feature corresponding to formate species was identified at 1606 cm^‒1^ ^61^, while it was absent with N_2_ purging on the surface of the catalyst (Supplementary Fig. 37b). This result suggested that the reaction intermediate was generated in the presence of H_2_ during the CO_2_ dissociation. Moreover, the simultaneous injection of CO_2_ and H_2_ also promoted the formation of formate intermediates along with the gaseous and adsorbed CO signal (Fig. 6c), which was consistent with the TPSR results. DFT calculations were performed to further explore the associative mechanisms by using the Ni_8_/TiO_2_ catalyst model (Supplementary Fig. 35). Here, the carboxyl and the formate pathways were both taken into account. As shown in Fig. 6d and Supplementary Figs. 38 and 39, we presented the energy profiles of these two reaction pathways. It could be seen that the rate-determining steps of these two pathways were the formation of COOH* (carboxyl pathway) and HCOOH* (formate pathway), and the corresponding energy barriers were 1.57 eV and 1.94 eV (carboxyl: TS1; formate: TS3), respectively, indicating that the carboxyl pathway was kinetically favorable. Notably, the COOH* intermediate could easily decompose into CO* and OH*, while on the contrary, HCOO* was difficult to further hydrogenated and converted into products. This suggested that COOH* was a more active intermediate than HCOO*, which was in consistent with the previously reported spectator role of formate^62^.

## Discussion

For heterogeneous catalytic reactions, the conversion of reactants and the selectivity of products often change largely along with the evolution of catalyst structures. Therefore, the construction of dense and stable active structures has always been at the core of the catalyst design. In this work, the ability of the commercial TiO_2_ to anchor active metals is greatly enhanced via the hydroxylation, which guarantees that highly loaded Ni species were anchored dominantly as single atoms. The strong interaction between intrinsic –OH groups and highly dispersed Ni species was proven to play an essential role in the formation of sintering-resisting clusters in the RWGS reaction. Due to the strong MSI between Ni clusters and TiO_2_ induced by the surface –OH, the formed rich Ni/TiO_2_ interfaces not only promoted the H_2_-assisted CO_2_ dissociation but also suppressed the formation of CH_4_, thereby breaking the activity-selectivity trade-off of the RWGS reaction. This work provides a strategy to design high-performance supported catalysts for heterogeneous reactions with harsh conditions by constructing hydroxylated oxides with enriched –OH groups.

## Method

### Preparation of TiO_2_ tube precursor

All of the chemicals applied to our experiments were of analytical grade and were used without further purification or modification. The TiO_2_ tube precursor was prepared by hydrothermal method in Teflon-lined stainless-steel autoclaves. For a typical synthesis of support, 28 g of NaOH (Sinopharm) was dissolved in 70 mL of deionized water (10 mol/L). After that, 2 g of anatase TiO_2_ (Macklin, particle size = 100 nm, denoted as TiO_2_-Ref1) was added into the above solution with stirring for 1 h at room temperature. Then the mixture was heated at 130 °C for 24 h. After the hydrothermal synthesis, the fresh precipitates were filtered and washed with deionized water and 2 L of 0.2 M HCl (Sinopharm) aqueous solution followed by deionized water. The resulting material was dried in air at 80 °C for 18 h and then ground in a mortar. Hereafter, the TiO_2_ tube precursor was denoted as TiO_2_-OH.

### Preparation of Ni catalysts

In a typical deposition-precipitation (DP) method, 0.5 g TiO_2_-OH powders were added to 25 mL deionized water at room temperature under vigorous stirring. 4.28 mL of 0.1 mol/L Ni(NO_3_)_2_·6H_2_O (Sinopharm) were added to the above suspension dropwise. The pH value of the solution was kept at ca. 9 with the assistance of Na_2_CO_3_ (Macklin) during the whole course. After stirring at room temperature for 30 min, the precipitates were further aged at room temperature for 1 h. Then, they were purified by suction filtration with deionized water (1 L) at room temperature. Finally, the product was dried at 70 °C for 10 h and then calcined in air at 600 °C for 4 h^24,63^. In our report, the nickel-titanium samples are denoted as *x*Ni/TiO_2_-OH, where *x* is the nickel content in weight percent. The referenced catalyst was prepared by the same method only except that the calcination temperature was 500 °C was denoted as 10Ni/TiO_2_-Ref1-500. The referenced catalyst was also prepared by the DP method as above just with the other purchased commercial TiO_2_ (denoted as TiO_2_-Ref1).

### Scanning transmission electron microscopy (STEM) characterization

High-angle annular dark-field (HAADF) STEM images, X-ray EDS spectra and elemental mappings, and EELS measurements were obtained from a Thermo Scientific Themis Z microscope equipped with a probe-forming spherical-aberration corrector at an operating voltage of 300 kV (Analytical Instrumentation Center of Hunan University).

### X-ray photoelectron spectroscopy (XPS)

The XPS measurements were carried out at a Thermo scientific ESCALAB Xi^+^ XPS spectrometer from Thermo Fisher. Quasi in situ XPS experiment was carried out on the same instrument. The spectrums of Ni 2*p*, Ti 2*p*, C 1*s*, and O 1*s* were obtained after 3 h of reaction in 15% CO_2_/30% H_2_/N_2_ atmosphere at 600 °C. The C 1*s* signal located at 284.8 eV was used to calibrate each spectrum for accurate binding energies^24^.

### Catalytic tests

The catalytic performance evaluation was tested in a fixed-bed flow reactor under a gas atmosphere of 23% CO_2_/69% H_2_/N_2_ (66.7 mL min^−1^, Deyang Gas Company, Jinan) at 1 bar total pressure^24^. Before activity test, 10 mg catalysts (20‒40 mesh) diluted with 90 mg inert SiO_2_ were activated by 5% H_2_/Ar at 600 °C for 0.5 h followed by switching to the feed gas for testing. The 10Ni/TiO_2_-Ref1-500 catalyst was activated by 5% H_2_/Ar at 500 °C for 0.5 h followed by switching to the feed gas for testing. The test temperature ranges from 400 °C to 600 °C. Before the analysis of gas products, the RWGS reaction needs to stabilize for 1 h at each test temperature. The gas products were analyzed by using an on-line gas chromatograph equipped with a thermal conductivity detector (TCD). CO_2_ conversion and CO selectivity were calculated using the following equations:

1 $${X}_{{{{\mathrm{CO}}}}_{2}}=\frac{{n}_{{{{\mathrm{CO}}}}_{2}}^{{{\mathrm{in}}}}-{n}_{{{{\mathrm{CO}}}}_{2}}^{{{\mathrm{out}}}}}{{n}_{{{{\mathrm{CO}}}}_{2}}^{{{\mathrm{in}}}}}\times 100\%\,$$

2 $${S}_{{{\mathrm{CO}}}}=\frac{{n}_{{{\mathrm{CO}}}}^{{{\mathrm{out}}}}}{{{{\mathrm{n}}}}_{{{\mathrm{CO}}}+}^{{{\mathrm{out}}}}{n}_{{{{\mathrm{CH}}}}_{4}}^{{{\mathrm{out}}}}}\times 100\%\,$$

where ${{{\mathrm{n}}}}_{{{{\mathrm{CO}}}}_{2}}^{{{\mathrm{in}}}}$ is the concentration of CO_2_ in the reaction stream, and ${{{\mathrm{n}}}}_{{{{\mathrm{CO}}}}_{2}}^{{{\mathrm{out}}}}$, ${{{\mathrm{n}}}}_{{{\mathrm{CO}}}}^{{{\mathrm{out}}}}$, ${{{\mathrm{n}}}}_{{{{\mathrm{CH}}}}_{4}}^{{{\mathrm{out}}}}$ are the concentrations of CO, CO_2_, CH_4_ in the outlet. For all catalysts, the *E*_*a*_ was measured by using the same reactor for catalytic performance above. Appropriate amounts of catalysts diluted with inlet SiO_2_ were used in the kinetics experiments. In order to obtain accurate kinetics data, the catalysts need to be first treated with reactive gas for an hour at 600 °C. During the kinetic test, the CO_2_ conversion remained between 5% and 15% by changing the gas flow rate. In order to minimize the effect of the external diffusion, for a reaction rate at 500 °C, 3 mg of catalyst and reaction gas flow of 120 mL/min were used, and CO_2_ conversion was 13.0% with CO selectivity of 98.07%. In order to better track the deactivation behavior, we used a much higher GHSV (800,000 mL g_cat_^−1^ h^−1^) in the long-term stability test. Due to the poor CO selectivity of the reference catalyst, the rate of CO_2_ conversion was used for the reaction rate at 400 °C, while the rate of CO production was used for the almost complete CO selectivity of the reference catalyst at 500 °C.

### Theoretical calculations

Details of the computational methods and the calculation model are put in the Supplementary Materials.

## Supplementary information

Supplementary Information

Peer Review File

## Source data

Source Data

## Data Availability

The main data supporting the findings of this study are available within the article and its Supplementary Information. Additional data are available from the corresponding authors upon request. Source data are provided with this paper.
