# Supplementary material for: Hydroxylated TiO2-induced high-density Ni clusters for breaking the activity-selectivity trade-off of CO2 hydrogenation
Source: Nat Commun. 2024 Sep 27;15:8290. doi: 10.1038/s41467-024-52547-4 (PMC11437244; doi:10.1038/s41467-024-52547-4)
Supplement: Supplementary file 1 — Supplementary Information [file 41467_2024_52547_MOESM1_ESM.pdf]

Supplementary Information for:

Hydroxylated TiO_2-induced high-density Ni clusters for breaking the activity-selectivity trade-off of CO_2 hydrogenation

Cong-Xiao Wang,[1,#] Hao-Xin Liu,[1,#] Hao Gu,[2,#] Jin-Ying Li,[1] Xiao-Meng Lai,[1] Xin-Pu Fu,[1] Wei-Wei Wang,[1] Qiang Fu,[3,4] Feng Ryan Wang,[2,*] Chao Ma,[5,*] Chun-Jiang Jia[1,*]

[1]Key Laboratory for Colloid and Interface Chemistry, Key Laboratory of Special Aggregated Materials, School of Chemistry and Chemical Engineering, Shandong University, Jinan, 250100, China.
[2]Department of Chemical Engineering, University College London, Roberts Building, Torrington Place, London WC1E 7JE, UK.
[3]Hefei National Research Center for Physical Sciences at the Microscale, University of Science and Technology of China, Hefei, 230026, China.
[4]School of Future Technology, University of Science and Technology of China, Hefei, 230026, China.
[5]College of Materials Science and Engineering, Hunan University, Changsha, 410082, China.
[#]These authors contributed equally: Cong-Xiao Wang, Hao-Xin Liu and Hao Gu.

*Corresponding Author. Email: jiacj@sdu.edu.cn; cma@hnu.edu.cn; ryan.wang@ucl.ac.uk

Table of Contents

Supplementary Methods

Supplementary Figures

Supplementary Tables

Supplementary References

Supplementary Methods:

Transmission electron microscopy (TEM): The low-magnification TEM images were taken from a JEOL JEM-1011microscope operated at 100 kV.

High-resolution transmission electron microscopy (HRTEM): The HRTEM images were recorded on a JEOL JEM-2100 F microscope. The ultra-thin carbon film-coated copper grid was used to prepare the TEM samples.

Attenuated total reflectance-infrared spectroscopy (ATR-IR): All spectra of samples were collected using a NICOLET iS50 ATR instrument from Thermo Fisher with a KBr beam splitter. The spectra data were collected in the range of 4000–400 cm^{-1} (resolution: 4 cm^{-1}).

X-ray diffraction (XRD): The X-ray diffraction (XRD) pattern was carried out on a PANalytical X'pert3 powder diffractometer (40 kV, 40 mA) using Cu Kα radiation (λ = 0.15418 nm). The powder samples were placed on a quartz-glass holder and the diffraction angles ranged from 10° to 90°.

Temperature programmed measurements: Temperature-programmed reduction by hydrogen (H_2-TPR) was carried on a Builder PCA-1200 instrument. 50 mg catalysts (20–40 mesh) were pretreated by 20% O_2/N_2 at 600 °C for 0.5 h and then purged with Ar. The final test was carried out in 5% H_2/Ar (30 mL·min^{-1}) from room temperature to 800 °C. Temperature-programmed desorption by carbon dioxide (CO_2-TPD) and Argon (Ar-TPD) were carried on the same instrument. The outlet gases were recorded by mass spectrum (MS-200). 50 mg catalysts were reduced in 5% H_2/Ar at 600 °C for 0.5 h, and then flushed with Ar gas flow (30 mL·min^{-1}) at room temperature for 0.5 h. For CO_2-TPD, the catalysts were saturated with 2% CO_2/Ar (30 mL·min^{-1}) at room temperature for 0.5 h followed by purging with Ar gas flow (30 mL·min^{-1}) for 0.5 h to clear away all the physical adsorbed CO_2 molecules. And then the CO_2-TPD experiment was started from room temperature to 800 °C with a ramping rate of 10 °C·min^{-1} under Ar gas flow (30 mL·min^{-1}). For Ar-TPD experiment, the catalysts were directly heated from room temperature to 800 °C under Ar atmosphere.

Ni K-edge X-ray adsorption fine structure spectroscopy (XAFS): The XAFS of Ni K-edge was carried out at beamline 01b1, SPring-8 (Super Photon ring-8) in Harima Science Garden City, Hyogo, Japan. The operation energy and ring current of storage ring were 8.0 GeV and 99.5 mA, respectively. Transmission mode was used and the monochromator was Si(111) and the staring angle and final angle were 14.3° and 11.9° to achieving the energy range from 8010 eV to 9582 eV. Data was processed by

the Software from Demeter package including Athena (version 0.9.26) and Artemis (0.8.012) based on Feff6.

Ni L-edge X-ray absorption spectroscopy (XAS): The XAS of Ni L-edge for 10Ni/TiO_2-OH catalyst was performed at beamline B07C, Diamond Light Source in Harwell Science & Innovation Campus, Didcot, Oxfordshire, UK. The sample was drop casting on Au coated Si wafer to enhance the conductivity. The data was processed by Dawn Diamond software and standards were measured for calibration of the energy shift. The XAS of L-edge for 10Ni/TiO_2-Ref1 was performed at beamline EMIL-CAT, BESSY II in Helmholtz-Zentrum Berlin, Berlin, Germany. Standards were measured for calibration of the energy shift. The sample was pressured into 5 mm pellet for measurement. The data was collected and processed by SpecsLab Prodigy developed by BESSY II.

CO_2 dissociation experiment, temperature-programmed surface reaction (TPSR): All these experiments were performed on a lab-made reactor. The outlet gases were recorded by mass spectrum (LC-D200M, TILON). 50 mg catalysts were pretreated in 5% H_2/Ar at 600 °C for 0.5 h and then purging with Ar at room temperature. For CO_2 dissociation experiment, the catalysts were started from room temperature to 600 °C with a ramping rate of 10 °C·min^{-1} under 2% CO_2/Ar atmosphere. For TPSR experiment, the catalysts were carried at the same way under 23% CO_2/69% H_2/N_2 atmosphere.

***In-situ* diffuse reflectance infrared Fourier transform spectroscopy (DRIFTS):** *In-situ* DRIFTS spectra were obtained using a Bruker Vertex 70 FTIR spectrometer fitted with an MCT detector. The DRIFTS cell (Harrick) was equipped with CaF_2 windows and a heating cartridge that allowed samples to be heated. An electro-control quick switching system was used to change the various gases rapidly to avoid dead volume as much as possible. About 10 mg of catalyst was tested in the reaction cell. Typically, an acquisition time of 30 s was used for spectrum collection at a resolution of 4 cm^{-1}. The background was obtained by using a spectrum recorded in pure N_2 at the same temperature as above. Before test, the sample was activated at 300 °C in 5% H_2/Ar (30 mL·min^{-1}) for 0.5 h, following under RWGS reaction at 300 °C for 0.5 h to make the interface similar to reaction state. Steady-state mode for all the catalysts was conducted in 15% CO_2/30% H_2/55% N_2 at 300 °C. In pulse mode, the gas was introduced to the catalyst by an order of 2% CO_2/N_2 – 5% H_2/Ar, 2% CO_2/N_2 – N_2 and 2% CO_2/N_2 – 15% CO_2/30% H_2/55% N_2.

***In-situ* infrared spectroscopy in the transmission mode.** *In-situ* infrared spectroscopy was carried out in a UHV apparatus by an FTIR spectrometer Bruker Vertex 70 (v) with a multichambered ultrahigh vacuum system. The samples were pretreated with H_2 in the sample cell at 600 °C and

dropped to −130 °C directly. Following that, adsorption was performed by passing 1 × 10^{-2} mbar CO accompanied by data collection. Then the sample cell was re-evacuated to 1x10^{-7} mbar and data were collected again. The similar experiment was also carried out after *in-situ* RWGS reaction at 600 °C.

Computational methods: All calculations were performed by the Vienna ab-initio simulation package (VASP) based on density functional theory (DFT).[1,2] The interaction between core and valence electrons was described by the project-augmented wave (PAW) method.[3] The plane wave basis sets with the energy cut-off value of 500 eV were used to solve the spin-polarized Kohn-Sham equations. Exchange-correlation energies were calculated via the generalized gradient approximation (GGA)[4] with the PBE functional. The DFT+U method adopting a simplified rotationally invariant formulation by Dudarev *et al.*[5] was applied to the calculation of the relevant properties of the TiO_2, and the effective U_{eff} values (U_{eff} = U - J) were set to 3.5 eV.[6,7] The first Brillouin zone was sampled using a 3 × 3 × 1 Monkhorst-Pack grid.[8] For all configurations optimized, the forces were converged below 0.03 eV/Å. The transition states were obtained by the climbing-image nudged elastic band method and the dimer methods,[9,10] and the forces were converged to below 0.1 eV/Å.

Calculation model: The optimized bulk lattice parameters were obtained by the DFT+U method**.** The most abundant anatase surface was the (101) termination.[11] Hence, TiO_2(101) surfaces were modeled by 3×1 supercell with three O-Ti-O layers. A vacuum layer of 15 Å thick was added along the [101] direction to eliminate the interaction between periodic images along the Z-axis. Compared with rutile, the oxygen vacancy on anatase surface was unstable and can migrate into the bulk, not to mention at a high temperature of 600 °C.[12,13] Therefore, surface oxygen vacancies were not considered in the simulation model. We explored many possible configurations of the Ni_8 clusters on the TiO_2(101) surface, and selected the most stable structure that could maintain the features of the original Ni_8 cluster to model the catalyst (Supplementary Fig. 35). Although we could not rule out the existence of more stable isomers of adsorbed Ni_8 owing to the complex and diverse surface configurations of metal clusters, we were confident that the currently employed model had reflected the key interactions between reactants/intermediates and the catalysts.

Supplementary Figures:

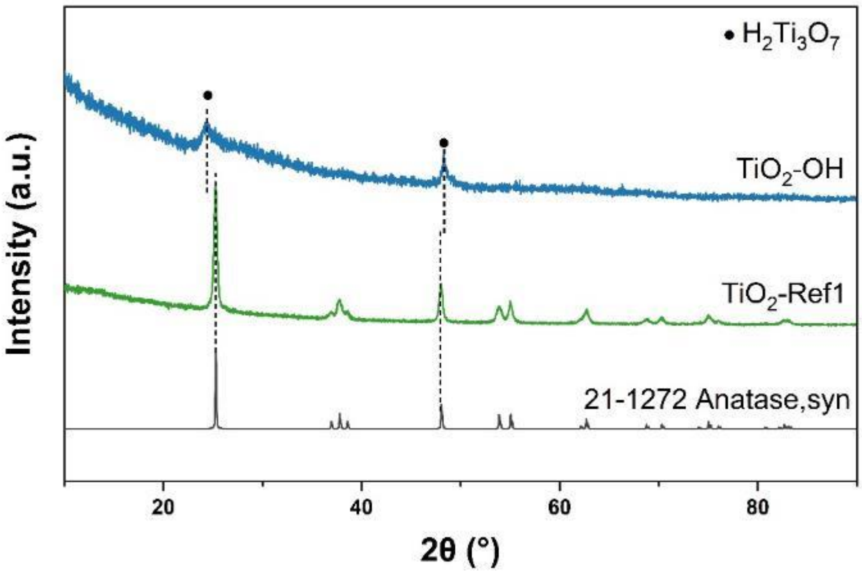

Supplementary Fig. 1 | The phase of different supports. XRD patterns of TiO_2-OH and TiO_2-Ref1.

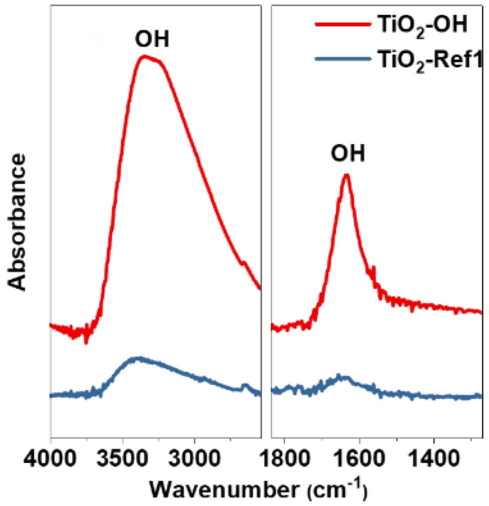

Supplementary Fig. 2 | The concentration of hydroxyl groups on the surface of the supports. The ATR-IR spectra of TiO_2-OH and TiO_2-Ref1 samples.

Supplementary Note 1: The ATR-IR spectra demonstrated that there were a large number of -OH species on the surface of TiO_2-OH compared to the commercial TiO_2.

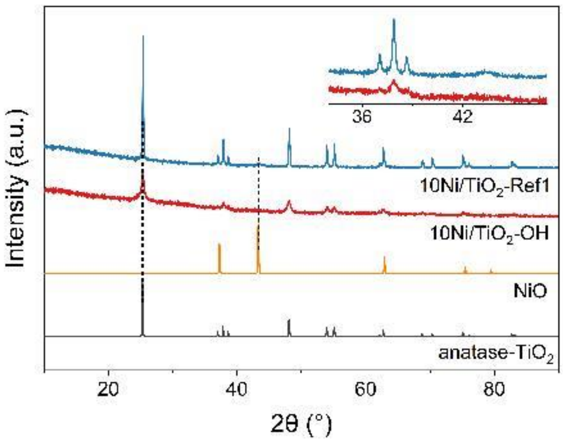

Supplementary Fig. 3 | The phase of different catalysts. XRD patterns of 10Ni/TiO_2-OH and 10Ni/TiO_2-Ref1 samples with the air calcination at 600 °C.

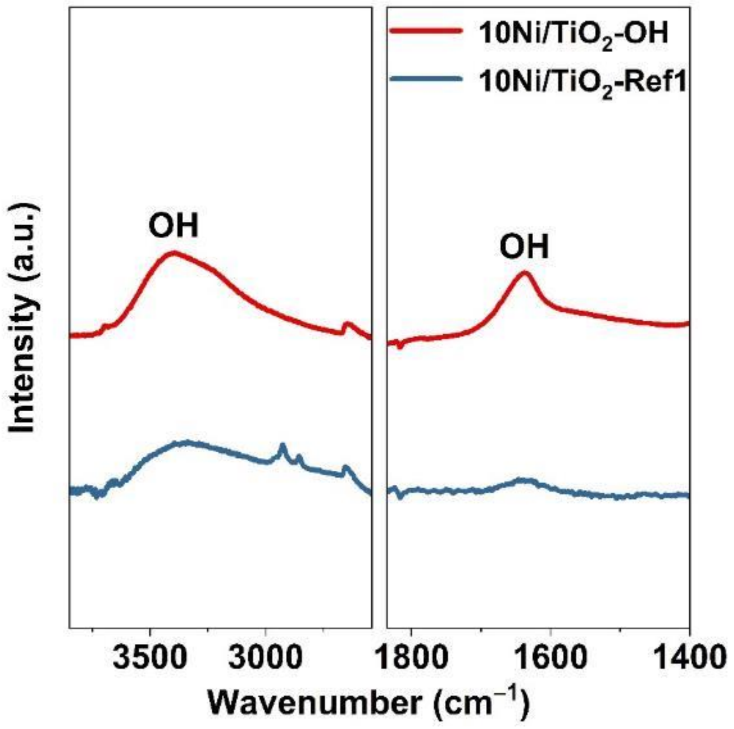

Supplementary Fig. 4 | The concentration of hydroxyl groups on the surface of the catalysts. The ATR-FTIR spectra of 10Ni/TiO_2-OH and 10Ni/TiO_2-Ref1 samples.

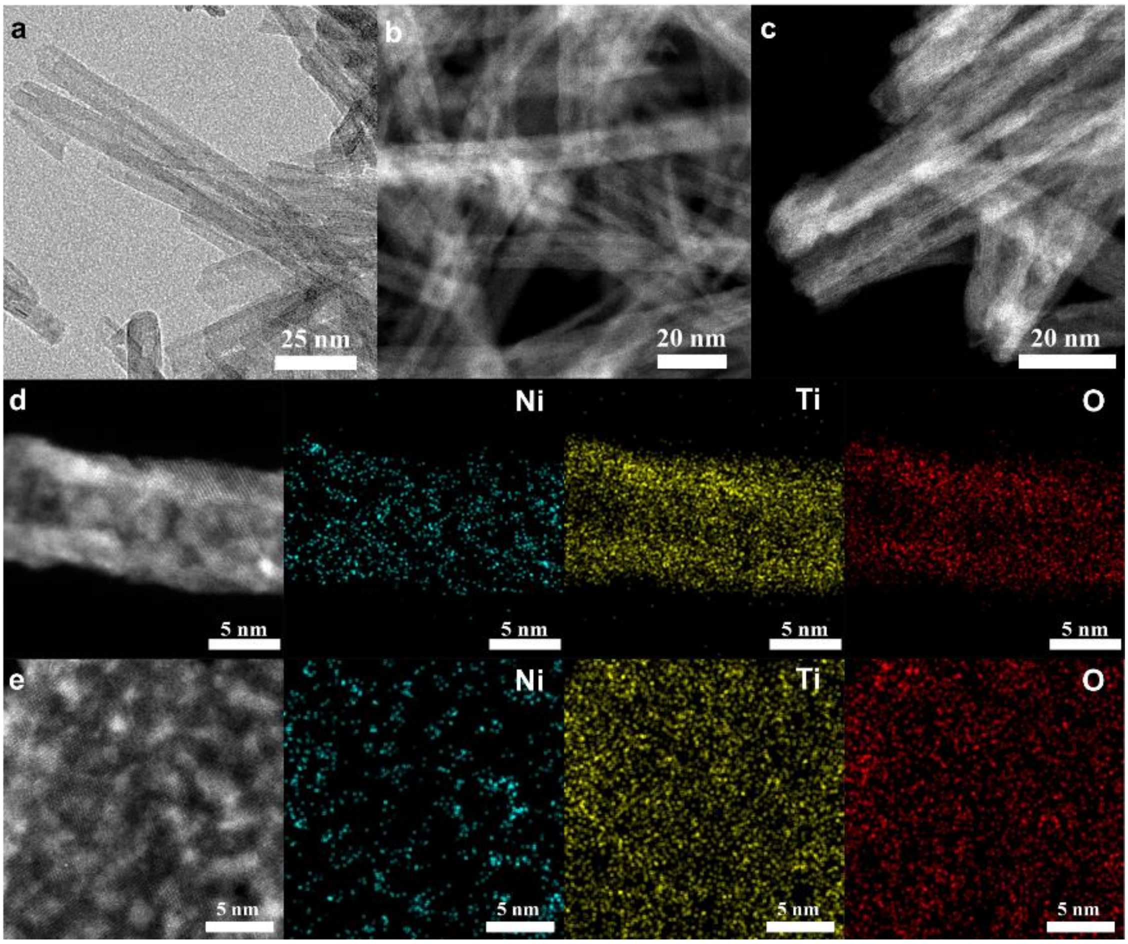

Supplementary Fig. 5 | The HRTEM and HAADF-STEM images of the 10Ni/TiO_2-OH catalyst. (a) HRTEM and (b, c) HAADF-STEM images of the 10Ni/TiO_2-OH catalyst. (d, e) EDS elemental mappings of the 10Ni/TiO_2-OH catalyst.

Supplementary Note 2: Ni species were highly dispersed on 10Ni/TiO_2-OH catalyst.

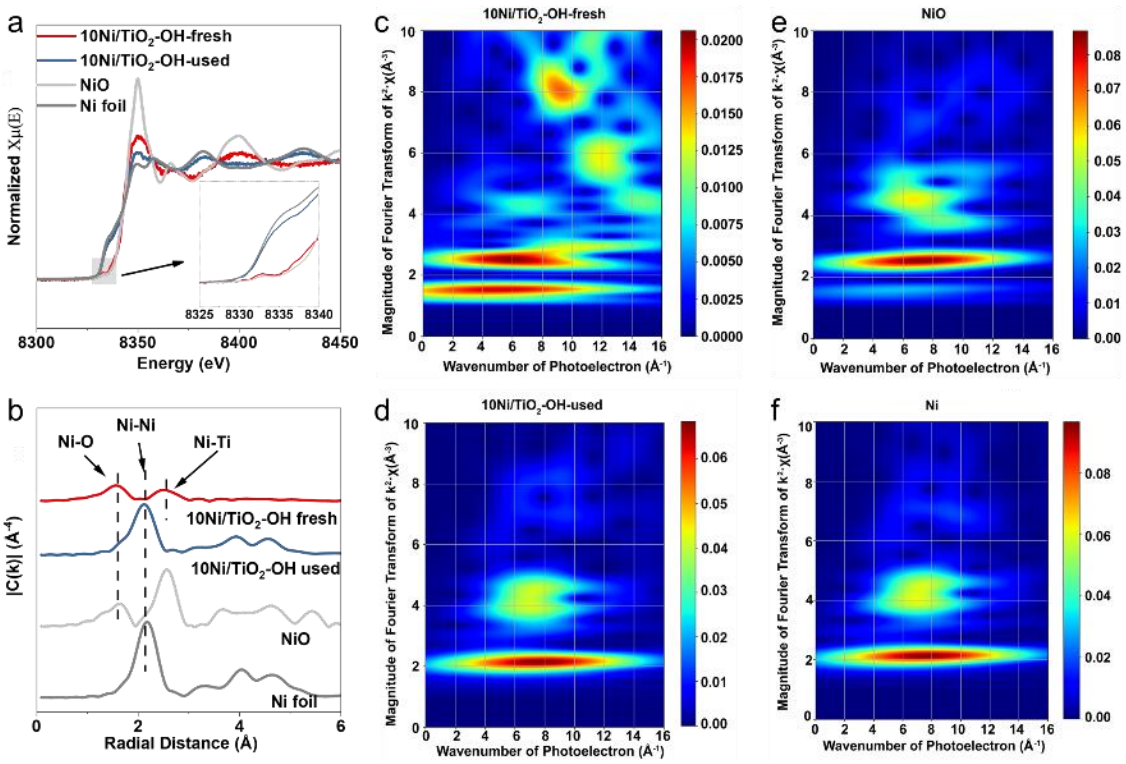

Supplementary Fig. 6 | X-ray absorption spectroscopy of the fresh and used 10Ni/TiO_2-OH catalysts. (a) X-ray absorption near edge spectroscopy (XANES) of the fresh and used 10Ni/TiO_2-OH catalysts. (b) Corresponded Extended X-ray absorption fine structure (EXAFS) profiles. 2-D contour plots wavelet transform results with experimental X(k) data of (c) the fresh, (d) used 10Ni/TiO_2-OH catalysts, (e) pure NiO and (f) Ni samples.

Supplementary Note 3: X-ray absorption fine structure spectroscopy (XAFS) for Ni K-edge was performed (fitting data: Supplementary Table 1). Typical Ni K-edge adsorption peak is observed at 8338 eV (Supplementary Fig. 6a). Compared with standards, the fresh catalyst showed similar feature to NiO, having a small pre-edge peak at around 8332 eV which indicating the transition of *1s* electron to *3d* orbital which became allowed transition because of the *3d*-*4p* orbital mixing.[14] After the reaction, the used catalyst was reduced and had similar features to Ni^0, which could be proved by higher intensity of pre-edge peak at 8334 eV and lower intensity of white line peak (*1s*-*4p*) at 8350 eV. The Fourier-transform EXAFS (Supplementary Fig. 6b) also showed the similar changing after reaction. However, the fresh catalyst only showed first and second shell coordination, which indicating the relative isolated distribution of Ni species. The feature peak at 1.6 Å was the Ni-O coordination and the feature peak at 2.5 Å could be Ni-Ti or Ni-Ni coordination which is difficult to confirm only by FT-EXAFS. By comparing the maximum intensity of second shell of standard NiO (7 $Å^{-1}$) and fresh catalyst (6 $Å^{-1}$)

in Wavelet transform (WT) EXAFS (Supplementary Fig. 6c–f), the conclusion was that the Ni-Ti coordination should be the second shell of fresh catalyst, which could also be the evidence of the large amount of highly dispersive Ni single site was formed. Ni-Ni was the main scattering features for used catalysts due to the formation of both clusters and big Ni particles.

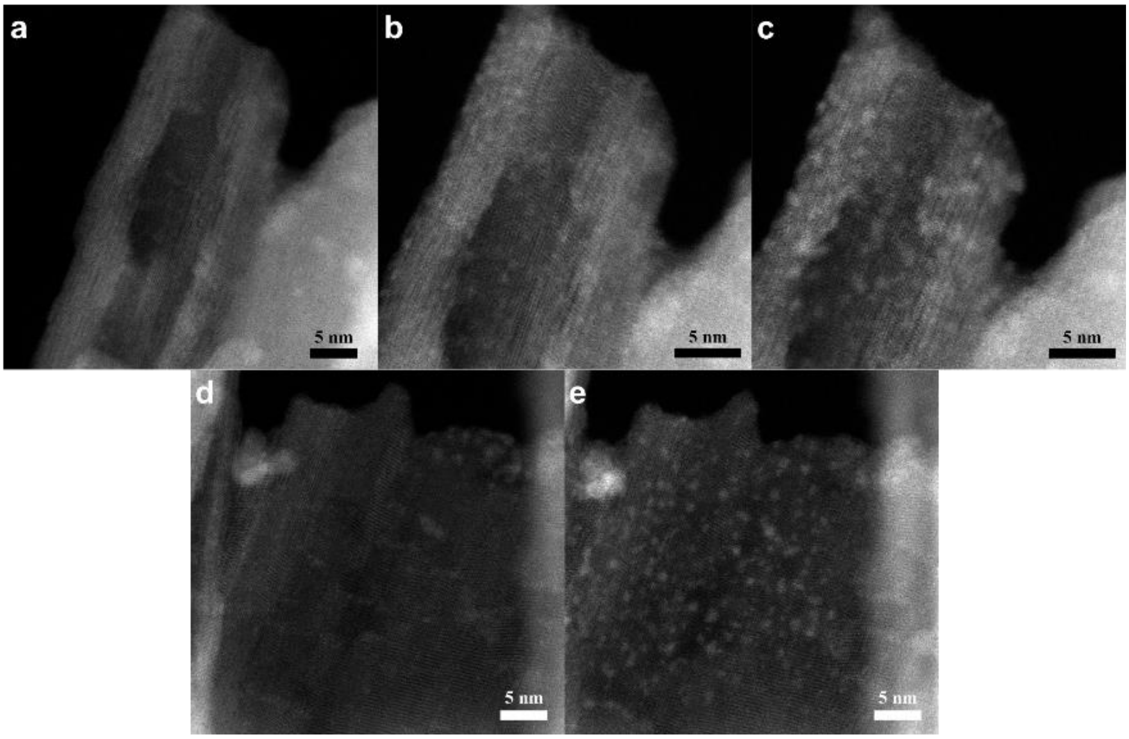

Supplementary Fig. 7 | HAADF-STEM images of Ni species affected by high-energy electrons over long time (60 s) in the 10Ni/TiO_2-OH catalyst. (a–c) Region 1 and (d, e) region 2.

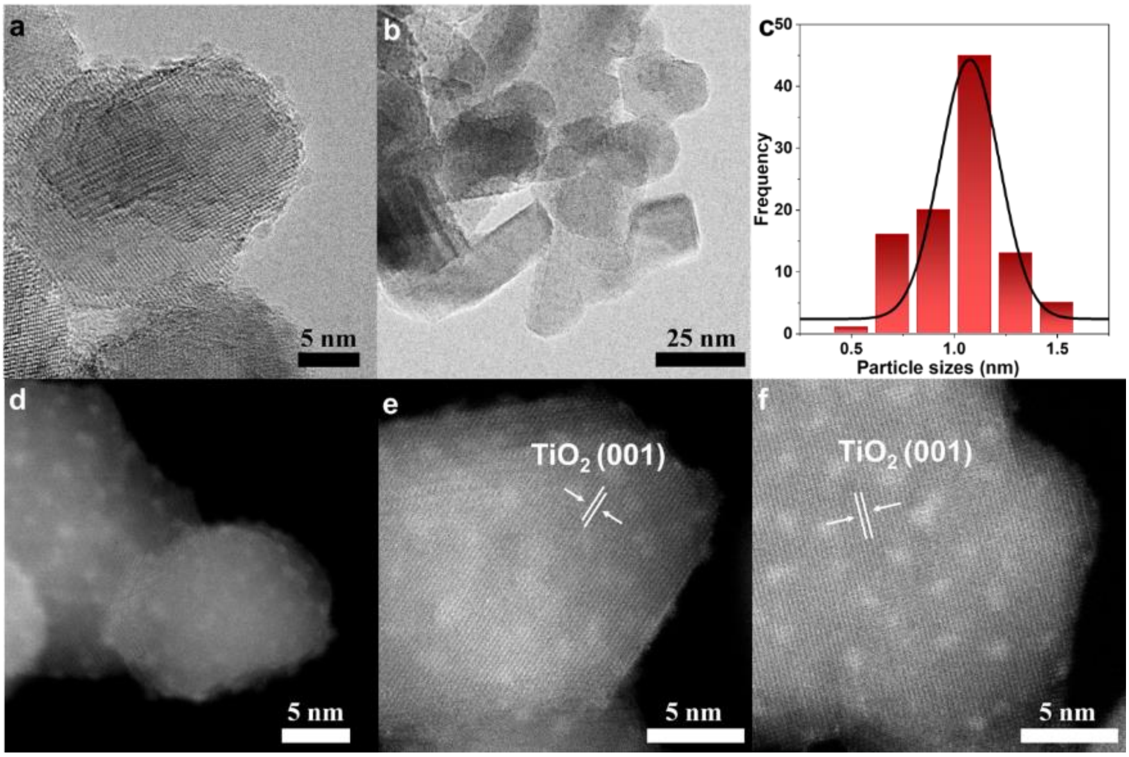

Supplementary Fig. 8 | The HRTEM images and HAADF-STEM images of the used 10Ni/TiO_2-OH catalyst. (a, b) HRTEM images. (c) Statistical histogram of size distribution of Ni clusters. (d–f) HAADF-STEM images.

Supplementary Note 4: After the RWGS reaction, part of Ni species turned into clusters with a particle size of about 1 nm for 10Ni/TiO_2-OH catalyst, which were highly dispersed on the TiO_2-OH support.

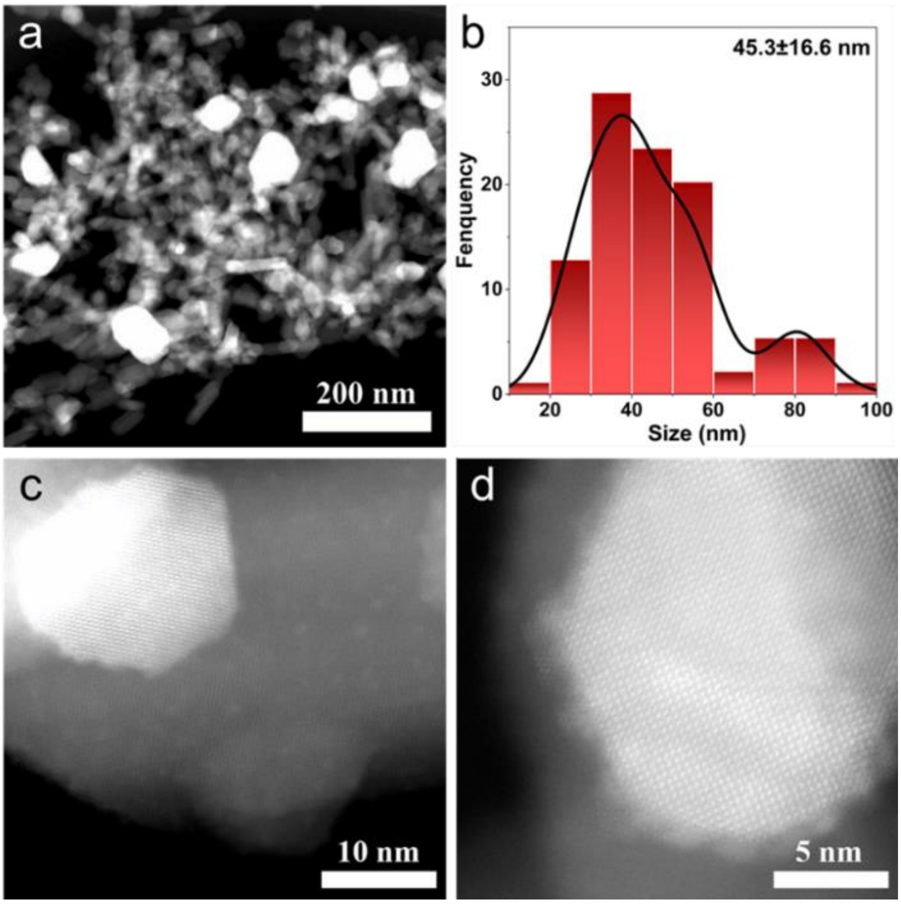

Supplementary Fig. 9 | The HAADF-STEM images of the used 10Ni/TiO_2-OH catalyst. (a) HAADF-STEM images over a wide range. (b) Statistical histogram of the size distribution of Ni particles. (c, d) HAADF-STEM images for Ni particles of local range.

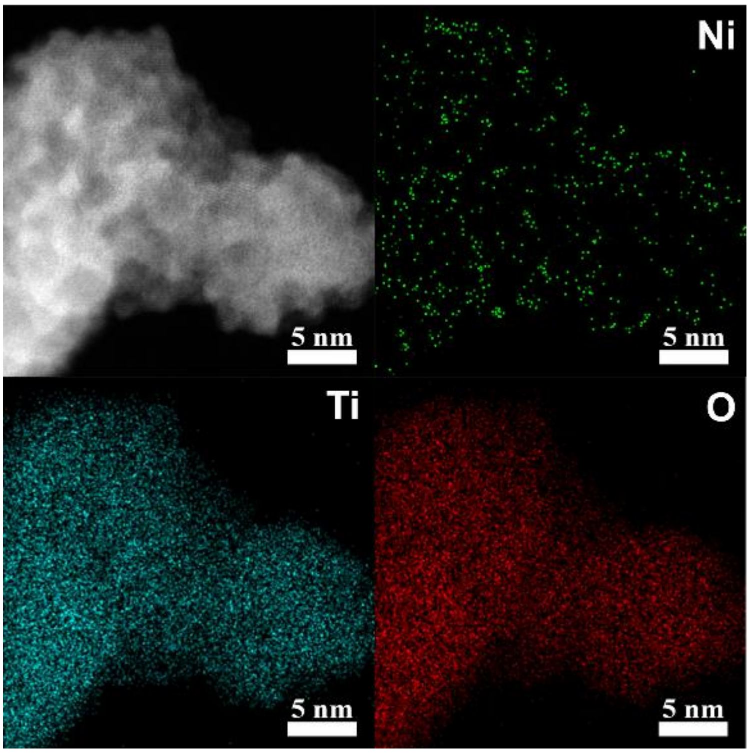

Supplementary Fig. 10 | The dispersion of Ni clusters over the used 10Ni/TiO_2-OH catalyst. EDS elemental mappings of Ni clusters over the used 10Ni/TiO_2-OH catalyst.

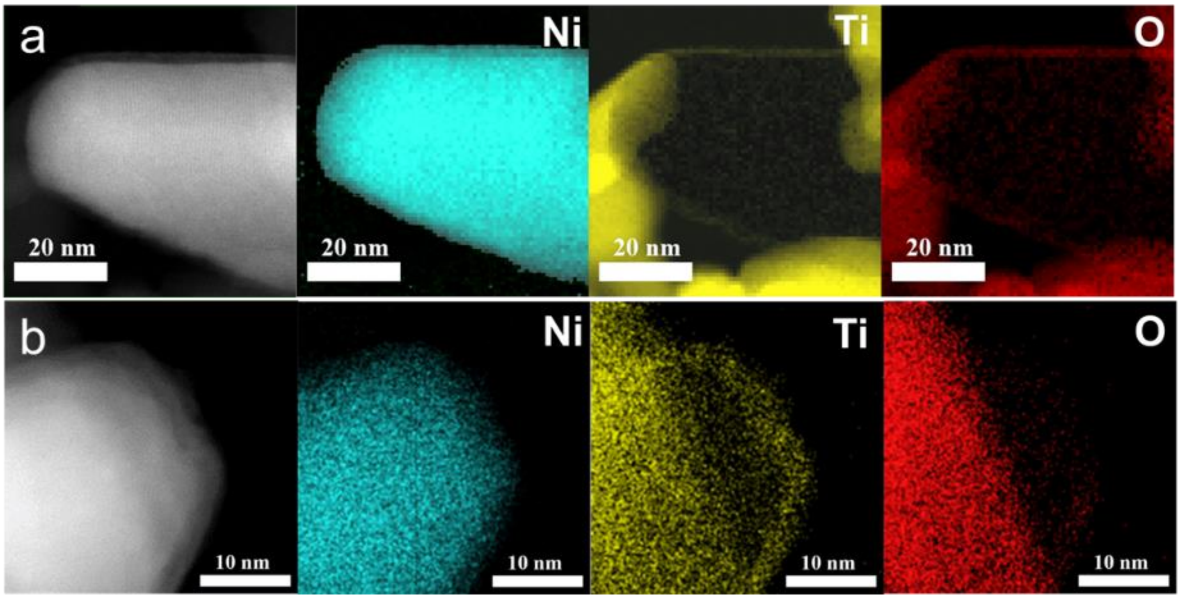

Supplementary Fig. 11 | EELS-mapping results for Ni particles of the used 10Ni/TiO_2-OH catalyst after the RWGS reaction. (a) Region 1 and (b) region 2.

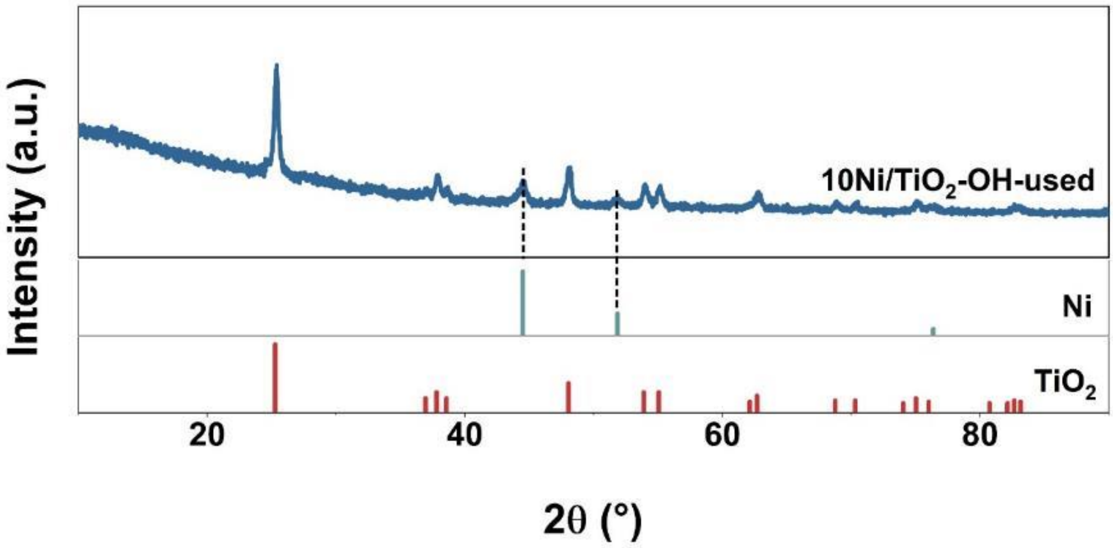

Supplementary Fig. 12 | The phase of 10Ni/TiO_2-OH catalyst after the RWGS reaction. XRD patterns of the used 10Ni/TiO_2-OH catalyst.

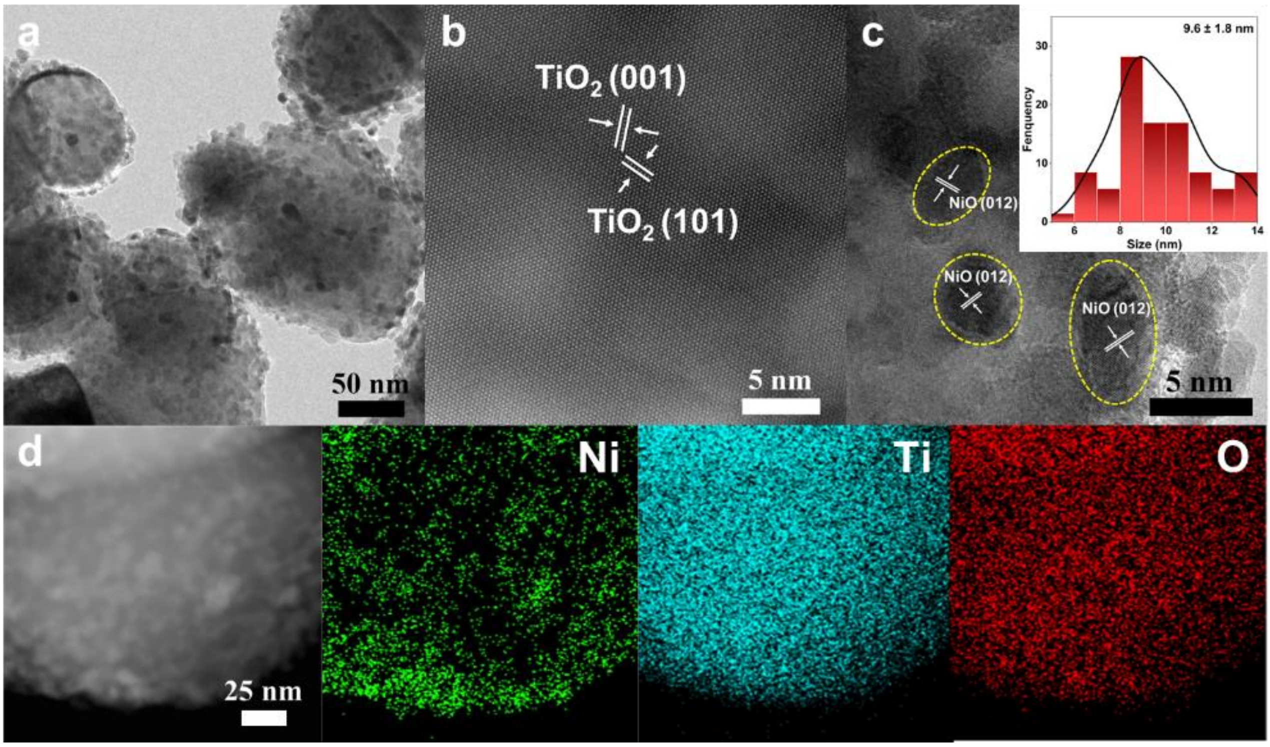

Supplementary Fig. 13 | The HRTEM image and HAADF-STEM images of the 10Ni/TiO_2-Ref1 catalyst. (a) The HRTEM image, (b, c) HAADF-STEM images and (d) EDS-mapping results. Inset: Statistical histogram of the size distribution of NiO particles.

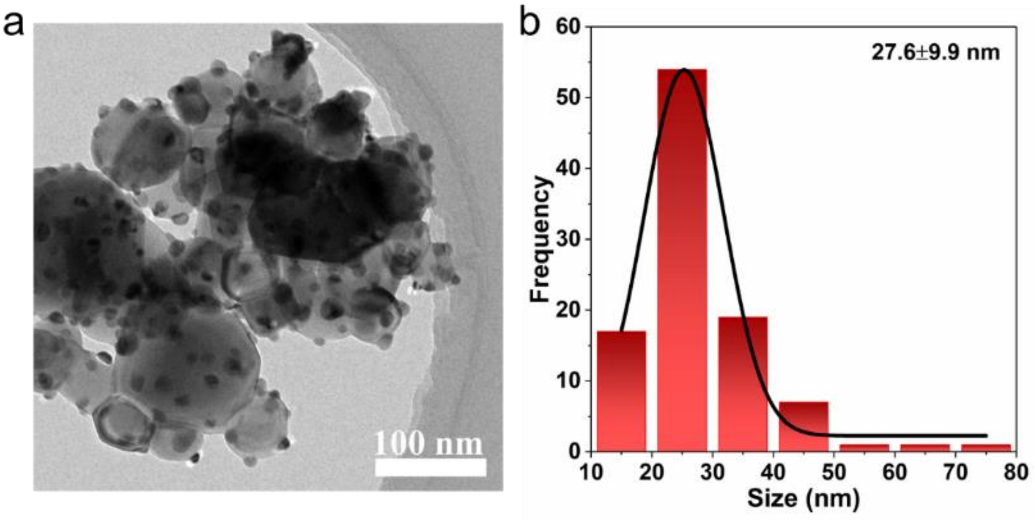

Supplementary Fig. 14 | The HETEM image and statistical analysis of particle size over 10Ni/TiO$_2$-Ref1 after the RWGS reaction. (a) The HETEM image. (b) Statistical analysis of particle size for Ni particles.

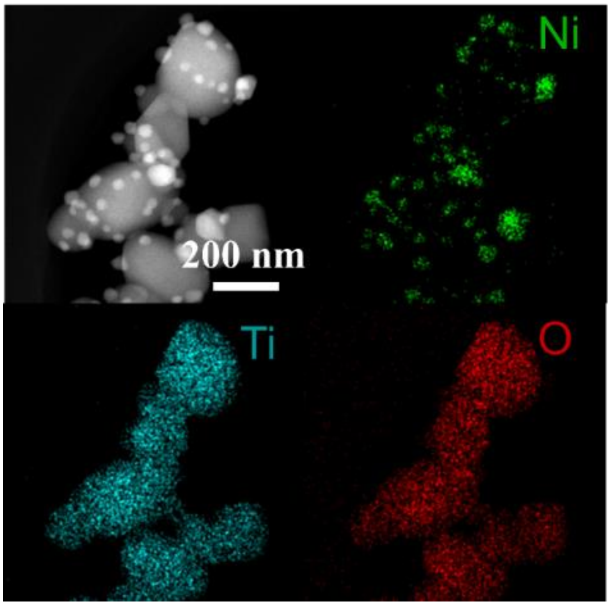

Supplementary Fig. 15 | The dispersion of Ni clusters over the used 10Ni/TiO_2-Ref1. HRTEM image of used 10Ni/TiO_2-Ref1 and corresponding EDS mapping results.

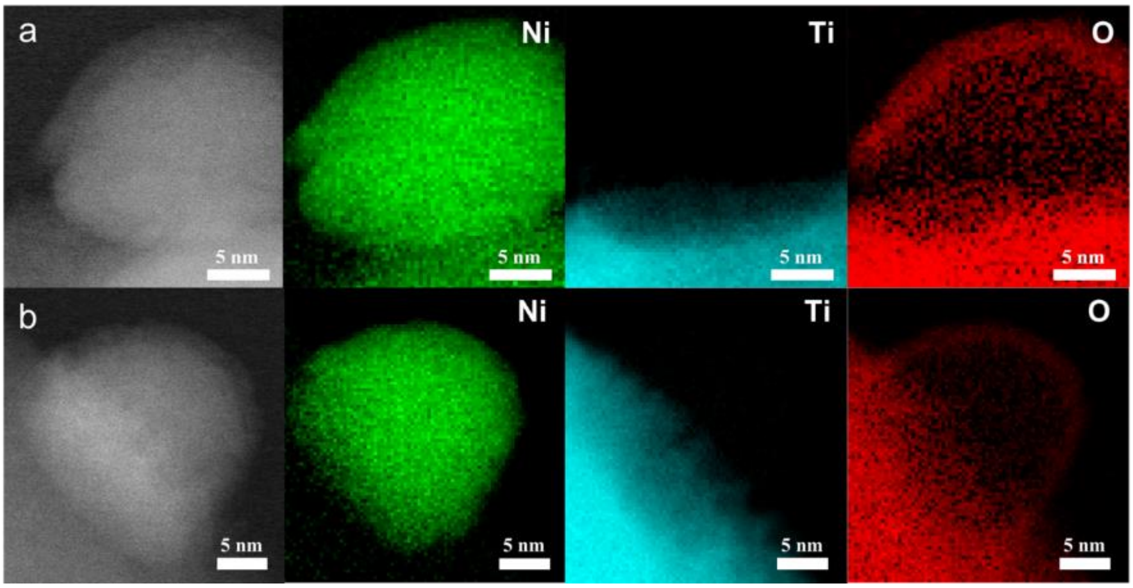

Supplementary Fig. 16 | EELS-mapping results of the used 10Ni/TiO_2-Ref1 catalyst. (a) Region 1 and (b) region 2.

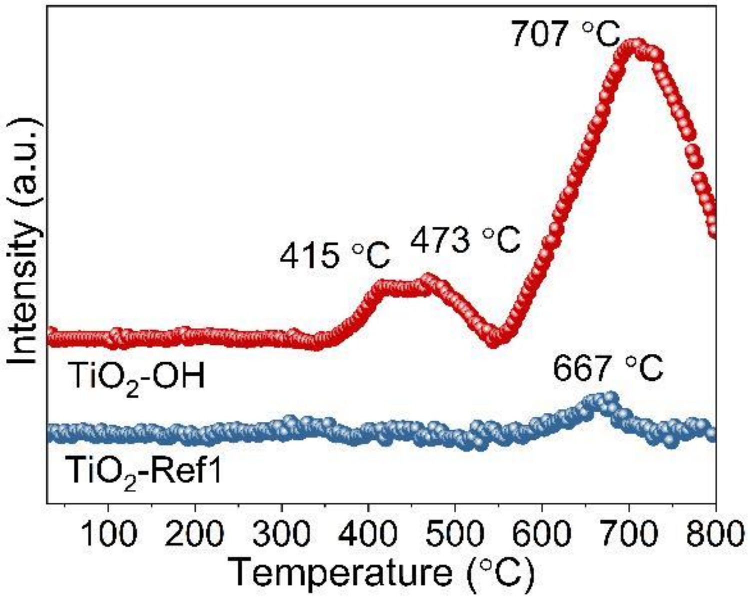

Supplementary Fig. 17 | The redox properties of different support. H_2-TPR results of TiO_2-OH and TiO_2-Ref1.

Supplementary Note 5: Compared to TiO_2-Ref1, TiO_2-OH showed stronger H_2 consumption peaks, suggesting much more oxygen atoms could be reduced by H_2 flow.

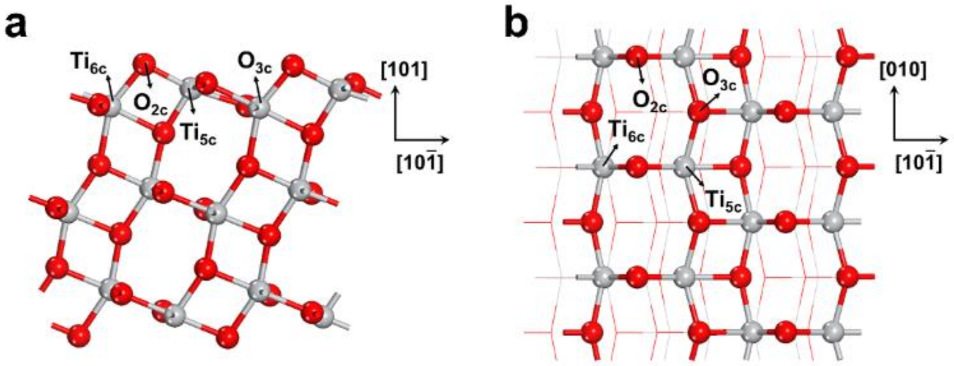

Supplementary Fig. 18 | Anatase (101) surface. (a) Front view and (b) top view.

Supplementary Note 6: Top view contains five-fold coordinated (Ti_{5c}) and six-fold coordinated (Ti_{6c}) Ti atoms, and three-fold coordinated (O_{3c}) and two-fold coordinated (O_{2c}) O atoms along the direction [010].

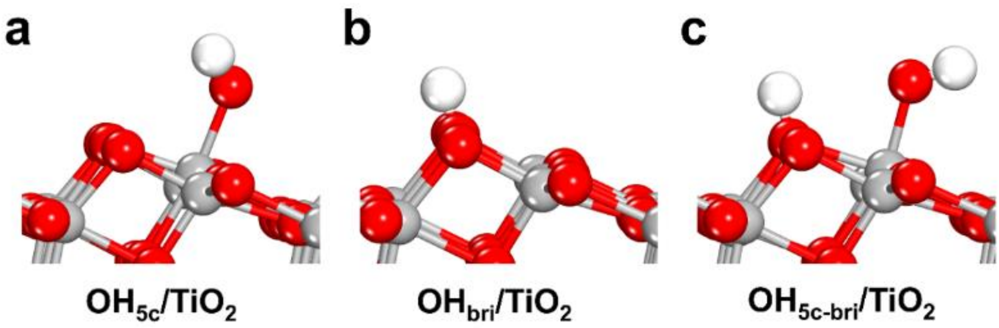

Supplementary Fig. 19 | Different hydroxylated TiO_2(101) surfaces. (a) A hydroxyl radical adsorbed to the Ti_{5c} site (OH_{5c}/TiO_2), (b) a hydrogen atom adsorbed to the bridging O_{2c} site (OH_{bri}/TiO_2), and (c) both adsorbed to on the surface ($OH_{5c\text{-}bri}/TiO_2$). The light gray, red and white spheres represent Ti, O, and H atoms, respectively.

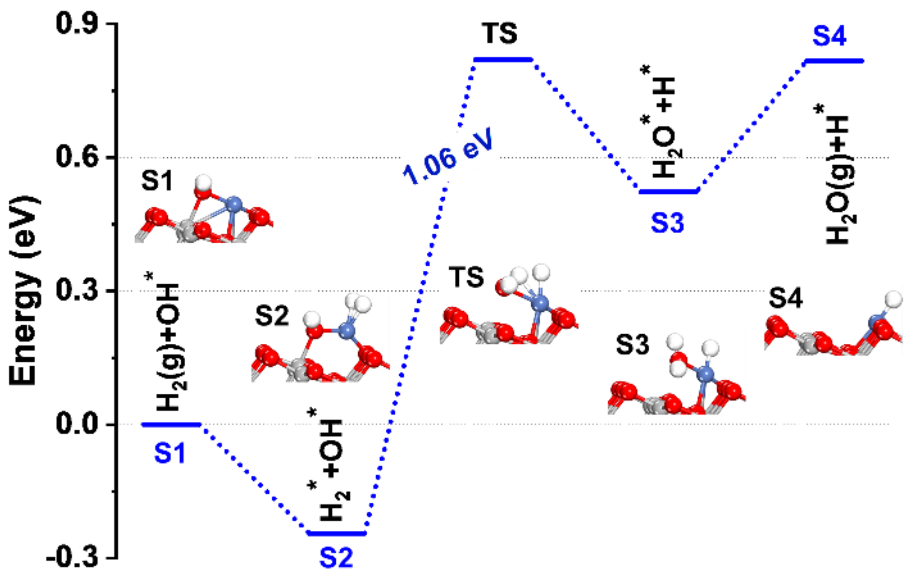

Supplementary Fig. 20 | Potential energy diagram for OH* to form H_2O* on TiO_2(101) surface, Ni/OH_{5c}-TiO_2-II is used as the initial model. TS represents the transition state. Color codes: light gray (Ti); red (O); blue (Ni); white (H).

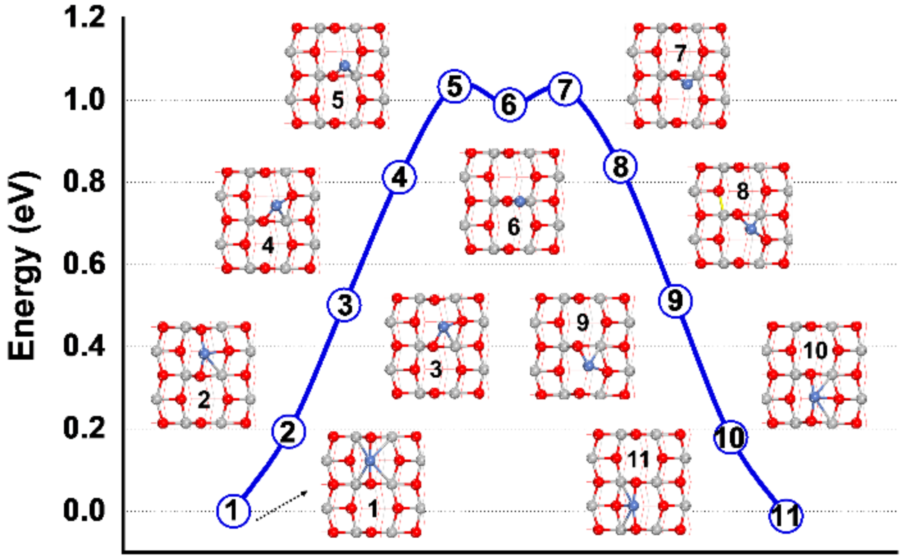

Supplementary Fig. 21 | The DFT result of the diffusion of Ni on TiO_2(101) surface. The diffusion potential energy diagram of Ni on TiO_2(101) surface.

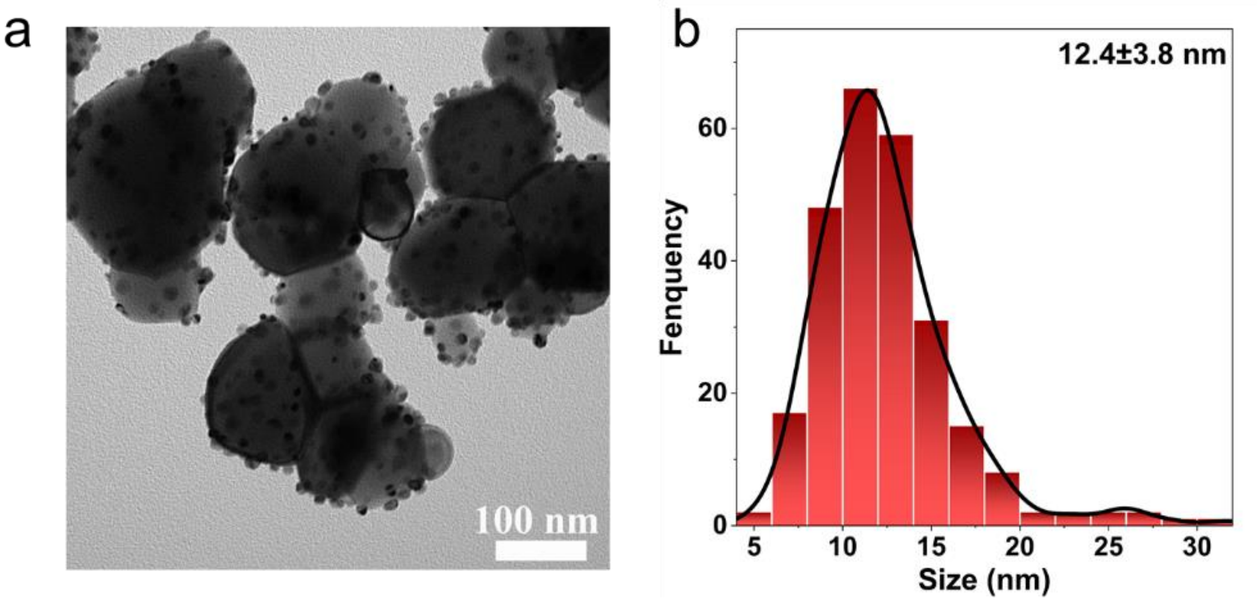

Supplementary Fig. 22 | The TEM image and the statistical analysis of particle size for 10Ni/TiO_2-Ref1 after air calcination and H_2 pretreatment at 500 °C. (a) The TEM image. (b) The statistical analysis of particle size for Ni particles.

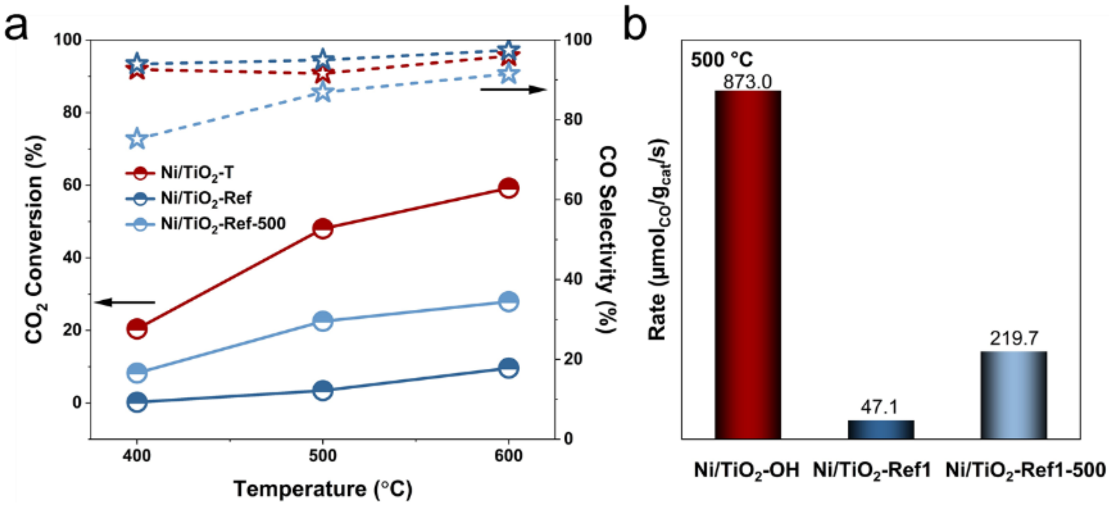

Supplementary Fig. 23 | Temperature-dependent activities of the catalysts under the GHSV of 400,000 mL·g_{cat}^{-1}·h^{-1}. (a) The CO_2 conversion and CO selectivity for 10Ni/TiO_2-OH and 10Ni/TiO_2-Ref1 after calcination and H_2 pretreatment at 600 °C, as well as 10Ni/TiO_2-Ref1 after calcination and H_2 pretreatment at 500 °C. (b) Comparison of CO yield rates over three samples at 500 °C.

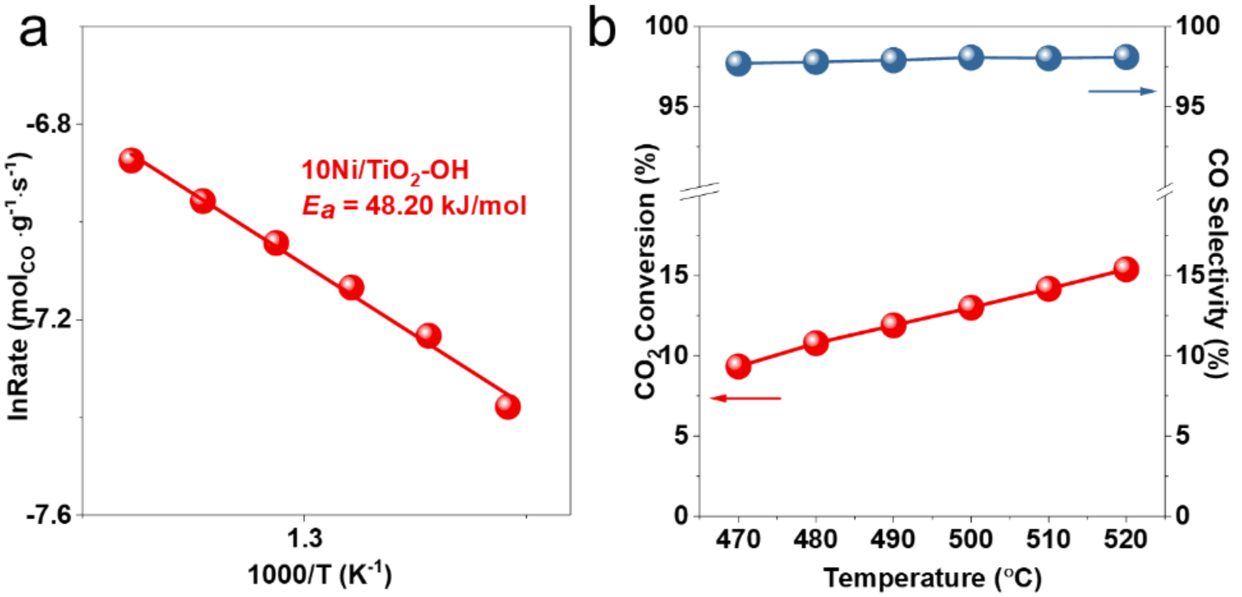

Supplementary Fig. 24 | Arrhenius plots for the catalysts of 10Ni/TiO2-OH. (a) The apparent activation energy (E_a). (b) CO_2 conversion and CO selectivity corresponding to the apparent activation energy in the kinetic interval.

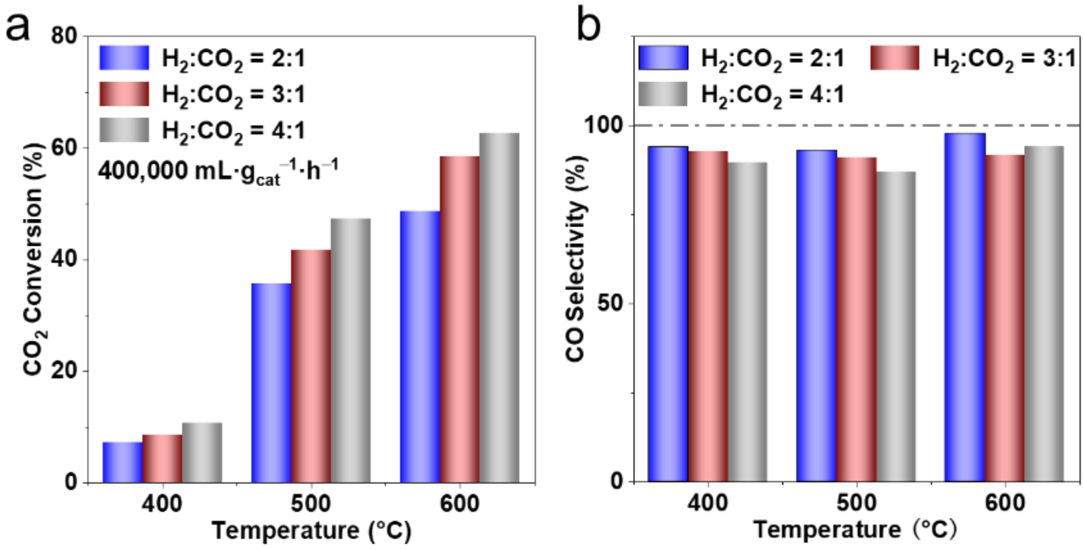

Supplementary Fig. 25 | Temperature-dependent activities of the catalysts for the 10Ni/TiO_2-OH catalyst at H_2:CO_2 ratios of 2:1, 3:1, and 4:1. (a) CO_2 conversion. (b) CO selectivity**.**

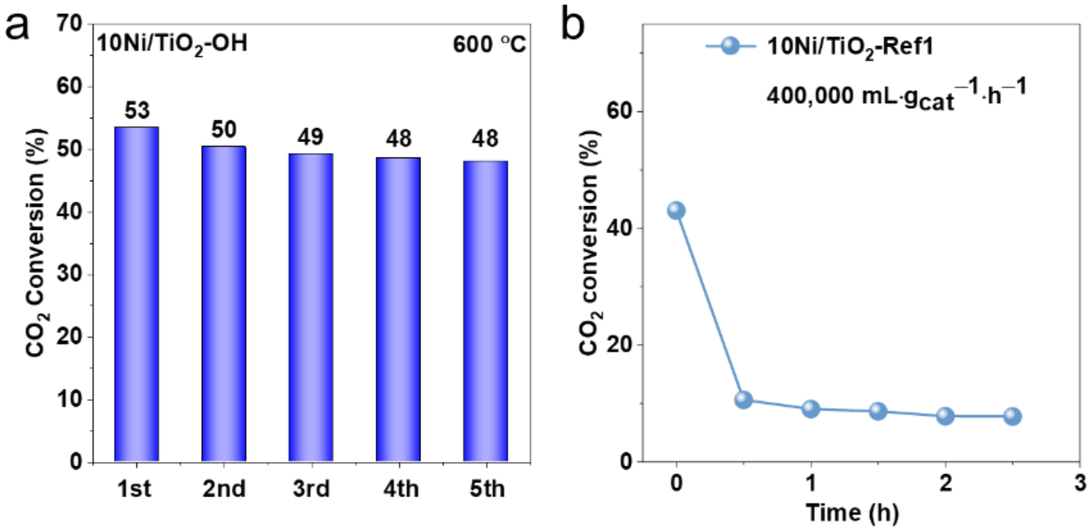

Supplementary Fig. 26 | The stability of 10Ni/TiO_2-OH and 10Ni/TiO_2-Ref1 catalyst. (a) CO_2 conversion over the 10Ni/TiO_2-OH catalyst for five start-up cool down cycles. (b) The long-time stability of the 10Ni/TiO_2-Ref1 catalyst at 600 °C.

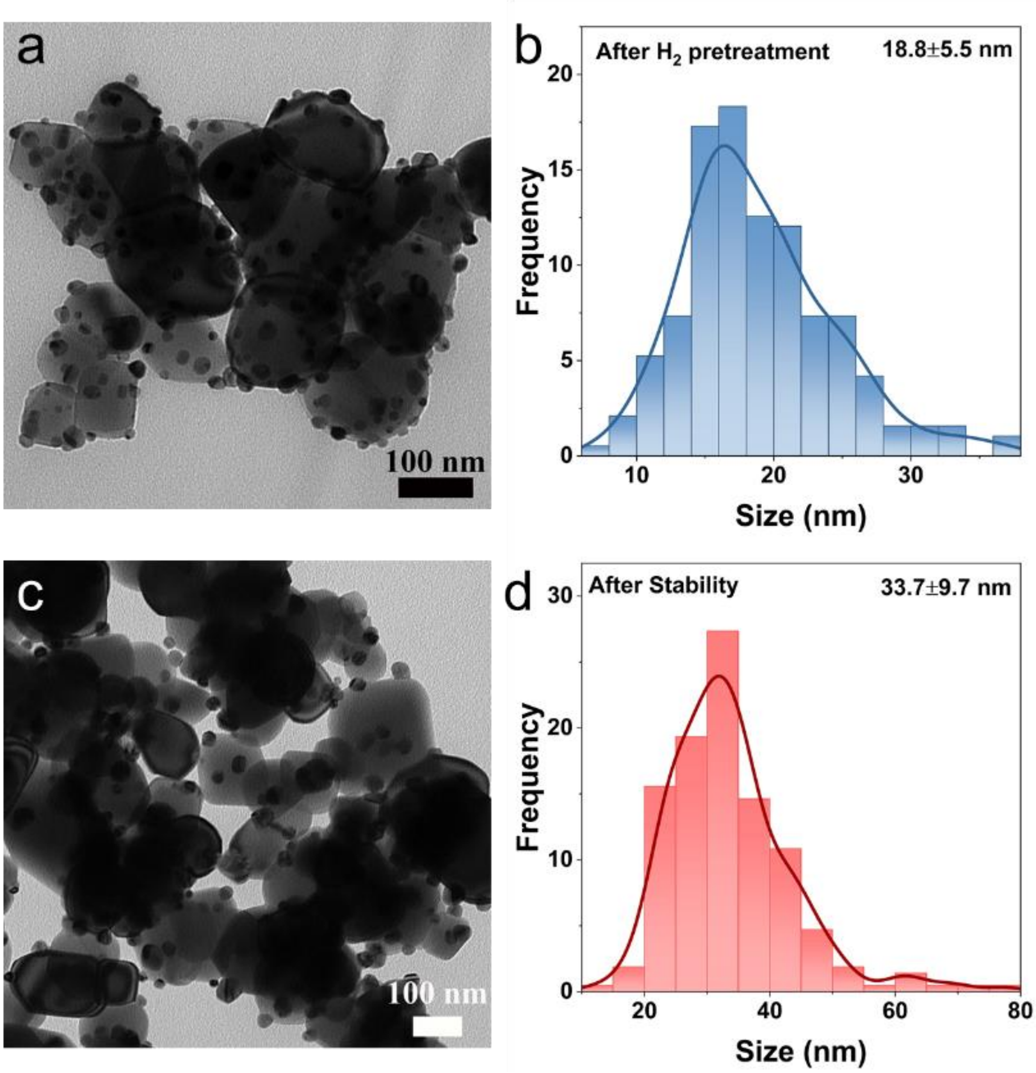

Supplementary Fig. 27 | TEM images and corresponding statistical analysis of particle size for Ni particles of 10Ni/TiO_2-Ref1 catalyst. (a, b) After H_2 pretreatment at 600 °C. (c, d) After long term stability test.

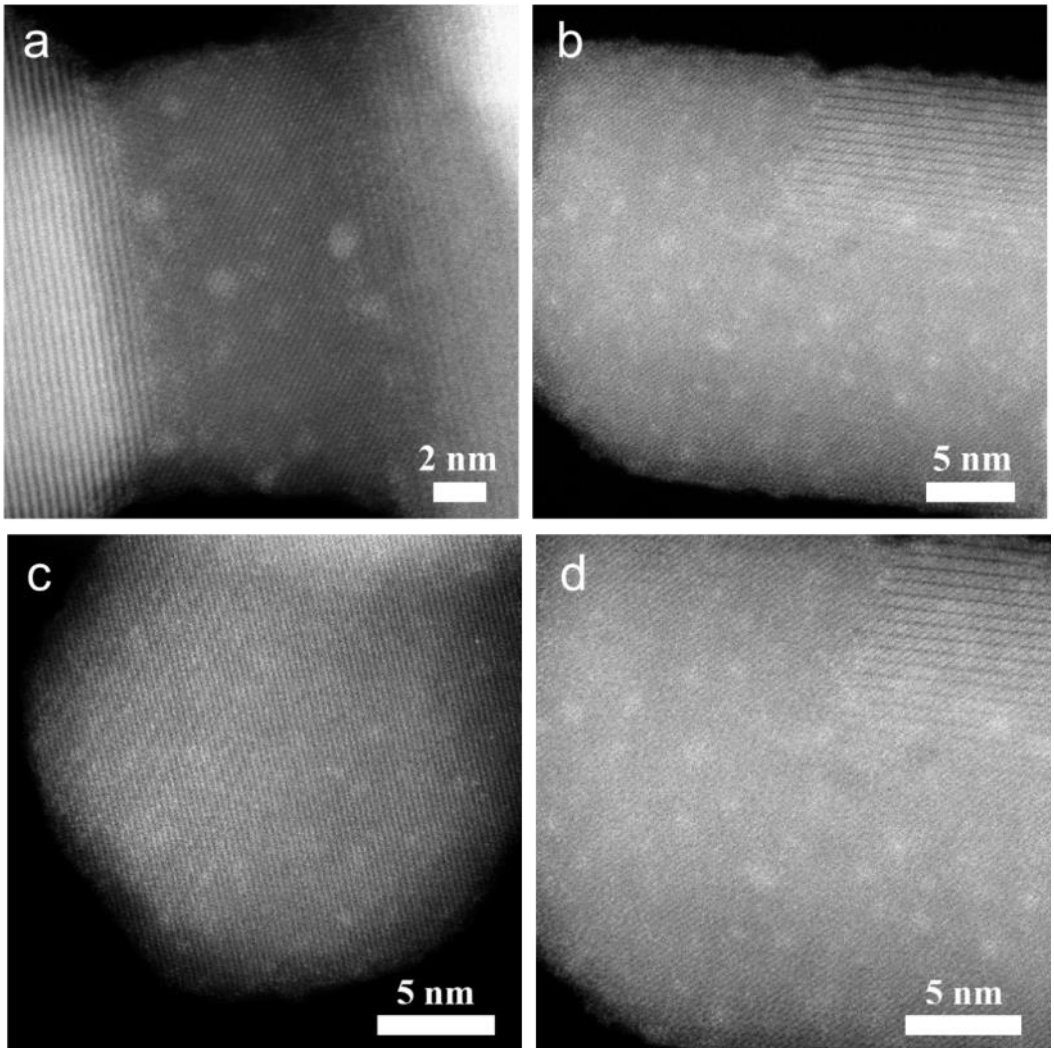

Supplementary Fig. 28 | HAADF-STEM images of the 10Ni/TiO_2-OH catalyst after 300 h stability test. (a) Region 1. (b) Region 2. (c) Region 3. (d) Region 4.

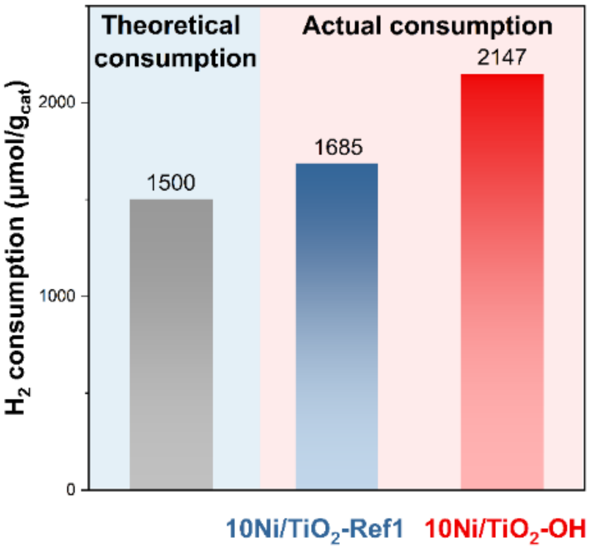

Supplementary Fig. 29 | H_2 consumption calculation from H_2-TPR results of 10Ni/TiO_2-OH and 10Ni/TiO_2-Ref1 in Fig. 5a. Theoretical consumption and actual consumption of 10Ni/TiO_2-Ref1 and 10Ni/TiO_2-OH catalyst.

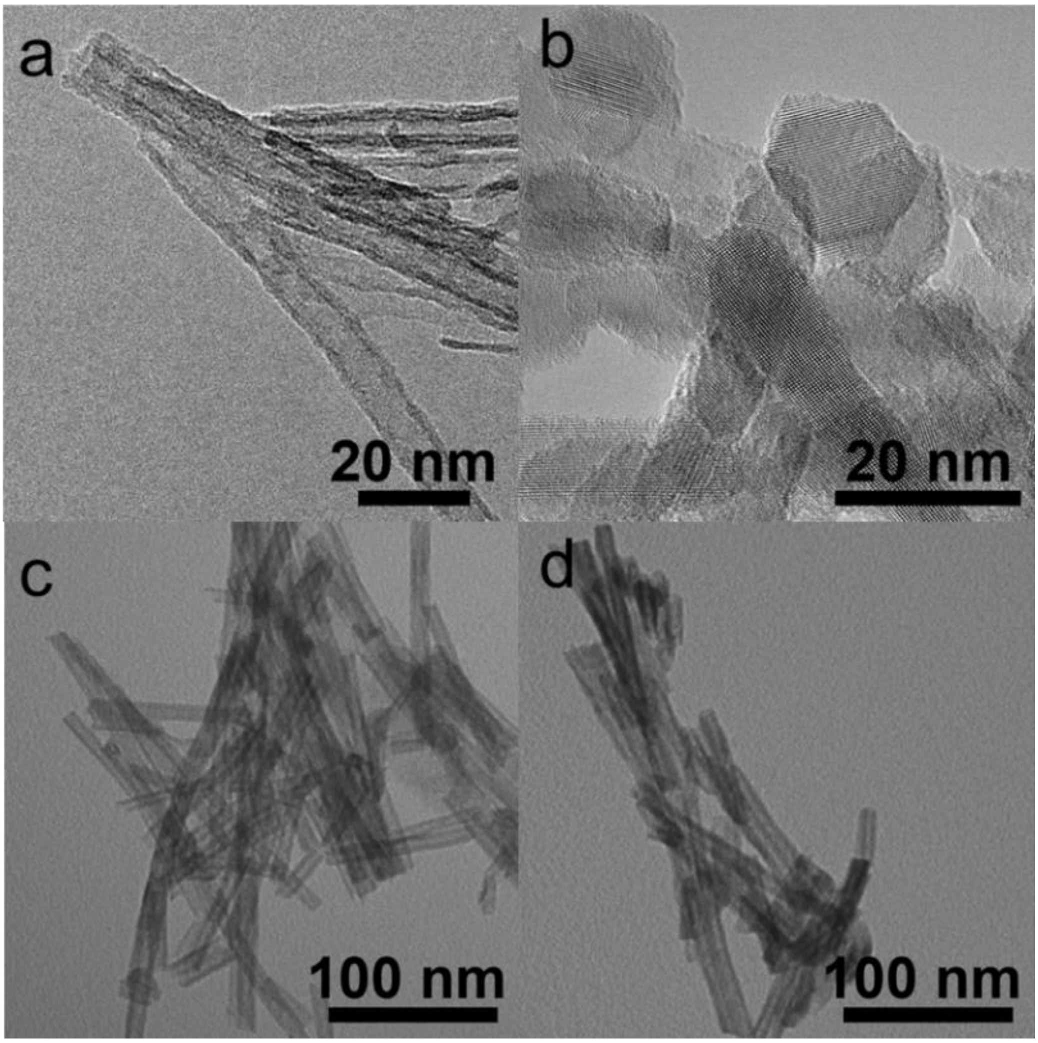

Supplementary Fig. 30 | The HRTEM images and TEM images. HRTEM images of (a) TiO_2-OH, and (b) TiO_2-OH samples after calcining at 500 °C. TEM images of (c) 10Ni/TiO_2-OH-UC and (d) 10Ni/TiO_2-OH.

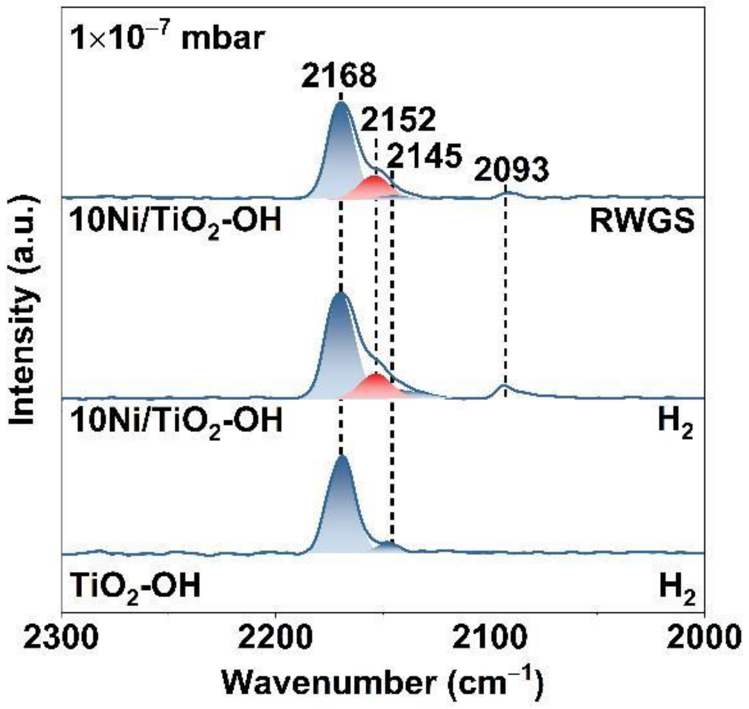

Supplementary Fig. 31 | *In-situ* infrared spectra of CO adsorption recorded at 130 K with 1x10^{-7} mbar. TiO_2-OH after H_2 pretreatment and 10Ni/TiO_2-OH catalysts after H_2 pretreatment and RWGS reaction.

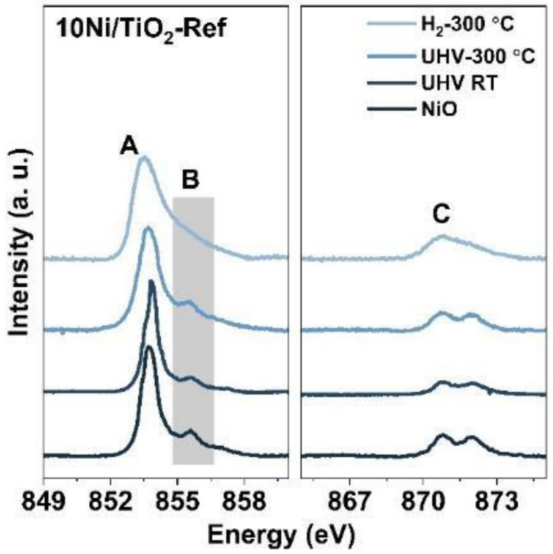

Supplementary Fig. 32 | The *in-situ* Ni L-edge NAP-NEXAFS profile of 10Ni/TiO_2-Ref1. Auger electron yield (AEY) for (A, B) Ni L3 (853 eV to 856 eV) and (C) L2 (870 eV to 872 eV) was observed, which were related to 2p3/2 to 3d and 2p1/2 to 3d transition, respectively.

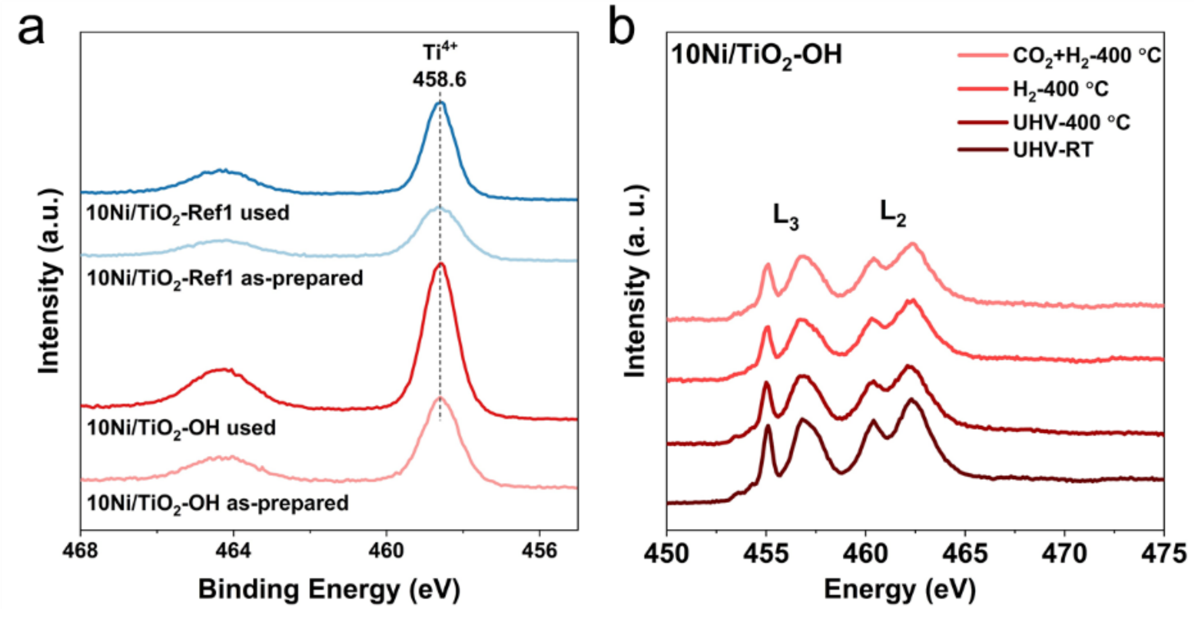

Supplementary Fig. 33 | *Ex-situ* XPS spectra and *in-situ* Ti L-edge NAP-NEXAFS profile. (a) XPS spectra of Ti *2p* in 10Ni/TiO_2-OH and 10Ni/TiO_2-Ref1 before and after the reaction. (b) *In-situ* Ti L-edge NAP-NEXAFS profile of the 10Ni/TiO_2-OH catalyst.

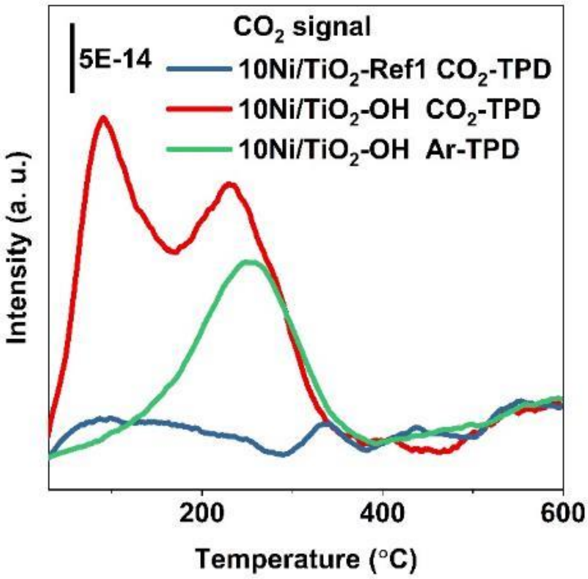

Supplementary Fig. 34 | The adsorption and activation capacity of CO_2 for different catalysts. CO_2-TPD profiles for 10Ni/TiO_2-Ref1 and 10Ni/TiO_2-OH catalysts and Ar-TPD profile for 10Ni/TiO_2-OH.

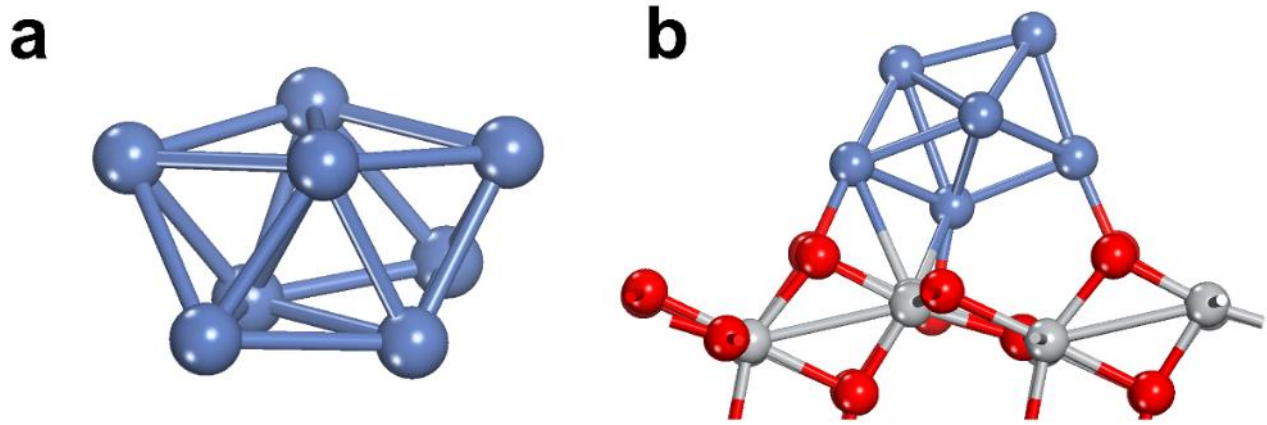

Supplementary Fig. 35 | Theoretical model of Ni_8 and Ni_8/TiO_2 configuration. (a) The most stable configuration of all the Ni_8 cluster configurations optimized. (b) The reasonable Ni_8/TiO_2 configuration from calculated configurations. Color codes: light gray (Ti); red (O); blue (Ni).

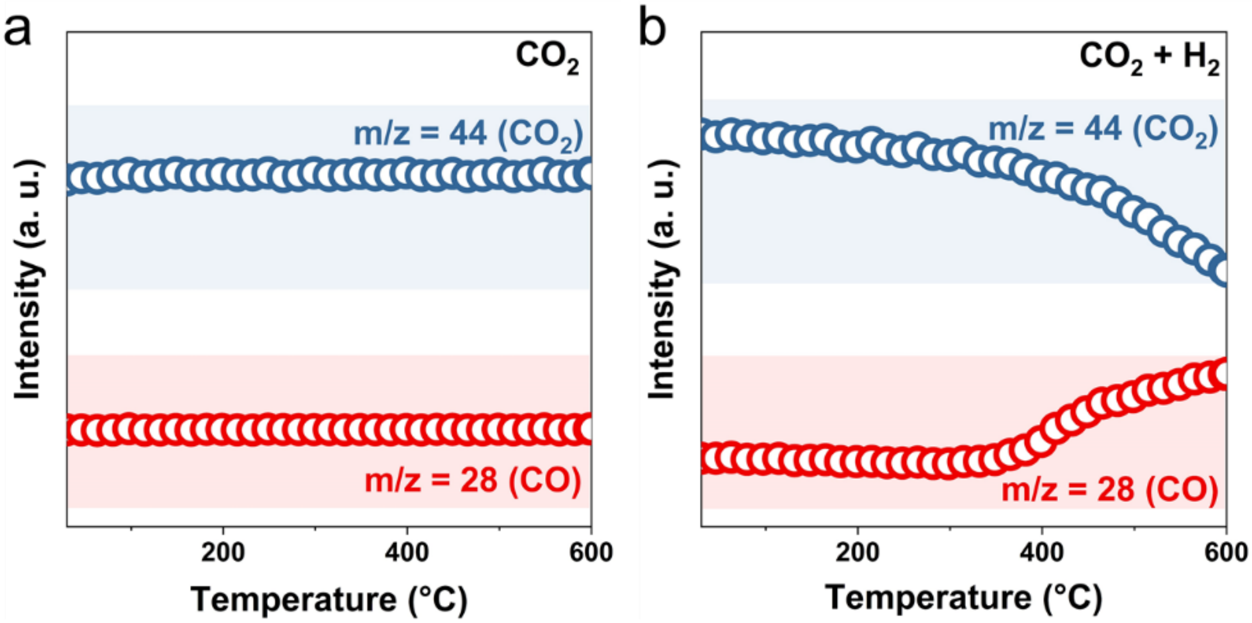

Supplementary Fig. 36 | The MS profiles of the 10Ni/TiO_2-OH catalyst. (a) CO_2 dissociation experiment. (b) TPSR results under CO_2+H_2 atmosphere.

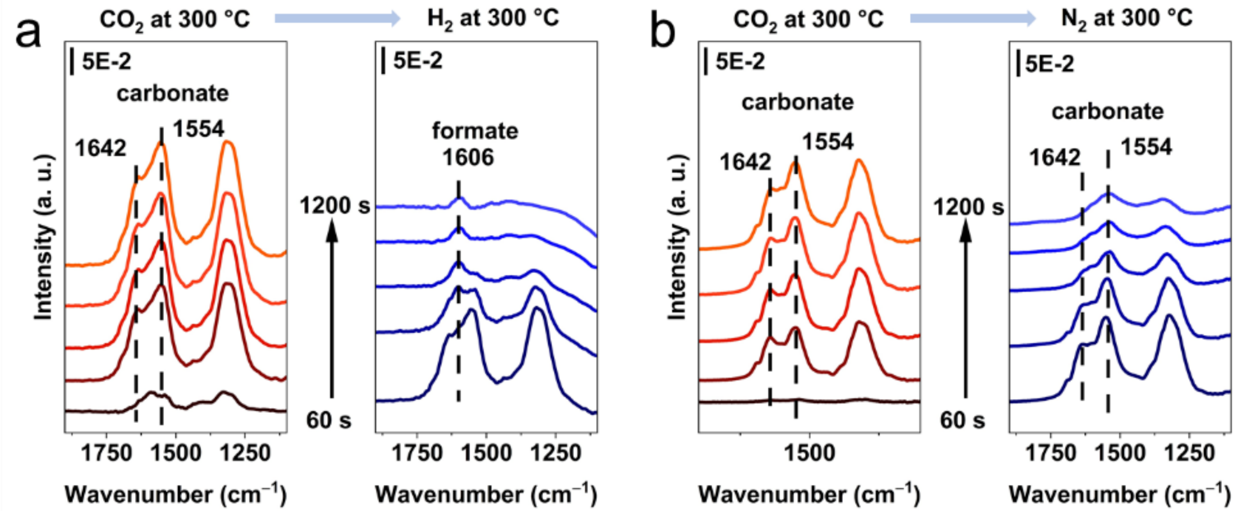

Supplementary Fig. 37 | *In-situ* DRIFTS spectra for the 10Ni/TiO_2-OH catalyst at 300 °C. (a) CO_2 adsorption → H_2 purging. (b) CO_2 adsorption → N_2 purging.

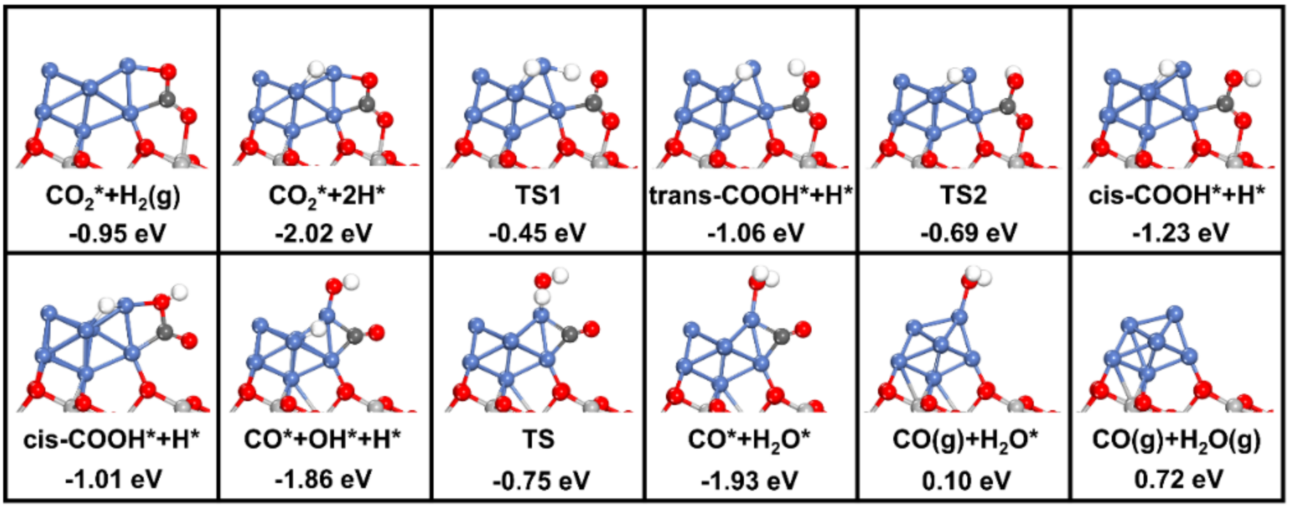

Supplementary Fig. 38 | The intermediates and transition states in carboxyl pathway. The energy value in the picture represents the relative energy based on the energy of Ni_8/TiO_2 configuration. Color codes: light gray (Ti); red (O); blue (Ni); white (H); grey (C).

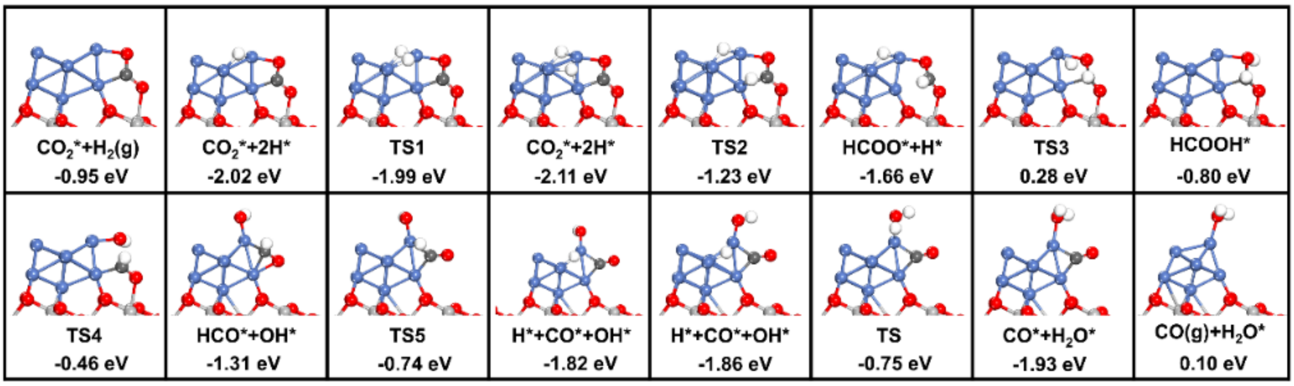

Supplementary Fig. 39 | The intermediates and transition states in formate pathway. The energy value in the picture represents the relative energy based on the energy of Ni_8/TiO_2 configuration. Color codes: light gray (Ti); red (O); blue (Ni); white (H); grey (C).

Supplementary Tables:

Supplementary Table 1. EXAFS fitting results of the fresh and used 10Ni/TiO_2-OH catalysts.

Catalysts				ΔE_0(eV)
10Ni/TiO_2-OH fresh	Ni–O			-2.947 ± 0.980
	R(Å)	CN	σ^2 (Å^2)	
	2.021 ± 0.009	3.316 ± 0.423	0.003 ± 0.001	
	Ni–Ti			
	R(Å)	CN	σ^2 (Å^2)	
	3.017 ± 0.013	2.692 ± 0.602	0.007 ± 0.002	
10Ni/TiO_2-OH used	Ni–Ni			-5.660 ± 1.120
	R(Å)	CN	σ^2 (Å^2)	
	2.485 ± 0.006	7.941 ± 0.737	0.005 ± 0.001	

Supplementary Table 2. Calculated adsorption energy and Bader charge of single-atom Ni on different surfaces.

Structure	Adsorption energy (eV)	Bader charge of Ni (|*e*|)	Bader charge of TiO_2 (|*e*|)
Ni/OH_{5c}-TiO_2-I	-5.86	+0.88	-0.88
Ni/OH_{5c}-TiO_2-II	-5.84	+0.78	-0.78
Ni/OH_{bri}-TiO_2	-2.95	+0.48	-0.48
Ni/OH_{5c-bri}-TiO_2	-3.18	+0.45	-0.45

Supplementary Table 3. Comparison of CO_2 conversion rate and CO selectivity for the as-prepared and literature reported catalysts.

Catalyst	H_2:CO_2	Temperature (°C)	Pressure (MPa)	Rate ($\mu mol_{CO}/g_{cat}/s$)	CO_2 Conv. rate ($\mu mol/g_{cat}/s$)	CO selectivity (%)	Ref
10Ni/TiO_2-OH	3:1	500	0.1	873.0[b]	—	98.0	this work
10Ni/TiO_2-OH	3:1	400	0.1	214.4[b]	233.3[b]	91.9	this work
(1) Ni/ MAO1000	4:1	400	0.1	—	53.3[a]	3	18
(2) 10Co/ r-TiO_2	4:1	400	3	—	19.16[a]	2	19
(3) Ni_3Fe_2 /ZrO_2	2:1	400	0.1	—	13.86[a]	11.5	20
(4) Ni_3Fe_3 /ZrO_2	2:1	400	0.1	—	13.57[a]	12.9	20
(5) Ni_3Fe_1 /ZrO_2	2:1	400	0.1	—	13.22[a]	14.0	20
(6) Ni_3/ZrO_2	2:1	400	0.1	—	12.6[a]	15.3	20
(7) Ni-P-12	4:1	400	0.1	—	1.83[a]	19.7	21
(8) 2Ni-4nm /CeO_2	3:1	400	0.1	—	74.4[b]	25.0	22
(9) Ni-P-8.7	4:1	400	0.1	—	74.4[a]	70.0	21
(10) Rh@S-1	3:1	400	1	—	3.87[a]	82.7	23
(11) 10Co/ a-TiO_2	4:1	400	3	—	3.33[a]	90	19
(12) Ni_3Fe_9 /ZrO_2	2:1	400	0.1	—	6.4[b]	95.8	20
(13) Ni-P-4.2	4:1	400	0.1	—	1.49[a]	96.6	21
(14) NiIn 0.5 /SBA-15	4:1	400	0.1	—	13.2[a]	99.0	24
(15) 2Ni-4nm /Mo_2N	3:1	400	0.1	—	134.0[b]	99.9	22

(16) Cu /β–Mo_2C	2:1	500	0.1	379.0[a]	—	99.0	25
(17) Cu /CeO_2–hs	3:1	500	0.1	334.7[a]	—	100	26
(18) Cu–Zn–Al	2:1	500	0.1	261.0[a]	—	100	26
(19) Ni in Cu	3:1	500	0.1	39.5[a]	—	100	27

[a]Out of the kinetic interval

[b]In the kinetic interval

Supplementary Refences:

1. Kresse, G. *et al.* Efficiency of ab-initio total energy calculations for metals and semiconductors using a plane-wave basis set. *Comput. Mater. Sci.* **6**, 15–50 (1996).
2. Kresse, G. *et al.* Efficient iterative schemes for ab initio total-energy calculations using a plane-wave basis set. *Phys. Rev. B* **54**, 11169–11186 (1996).
3. Blöchl, P. E. Projector augmented-wave method. *Phys. Rev. B* **50**, 17953–17979 (1994).
4. Perdew, J. P. *et al.* Generalized gradient approximation made simple. *Phys. Rev. Lett.* **77**, 3865–3868 (1996).
5. Dudarev, S. L. *et al.* Electron-energy-loss spectra and the structural stability of nickel oxide: An LSDA+U study. *Phys. Rev. B* **57**, 1505–1509 (1998).
6. Li, Y. *et al.* Interplay between water and TiO_2 anatase (101) surface with subsurface oxygen vacancy. *Phys. Rev. Lett.* **112**, 206101 (2014).
7. Setviń, M. *et al.* Reaction of O_2 with subsurface oxygen vacancies on TiO_2 anatase (101). *Science* **341**, 988–991 (2013).
8. Monkhorst, H. J. *et al.* Special points for Brillouin-zone integrations. *Phys. Rev. B* **13**, 5188–5192 (1976).
9. Henkelman, G. *et al.* A climbing image nudged elastic band method for finding saddle points and minimum energy paths. *J. Chem. Phys.* **113**, 9901–9904 (2000).
10. Henkelman, G. *et al.* A dimer method for finding saddle points on high dimensional potential surfaces using only first derivatives. *J. Chem. Phys.* **111**, 7010–7022 (1999).
11. Lazzeri, M. *et al.* Erratum: Structure and energetics of stoichiometric TiO_2 anatase surfaces. *Phys. Rev. B* **65**, 119901 (2002).
12. He, Y. *et al.* Evidence for the predominance of subsurface defects on reduced anatase TiO_2(101). *Phys. Rev. Lett.* **102**, 106105 (2009).
13. Scheiber, P. *et al.* (Sub)Surface mobility of oxygen vacancies at the TiO_2 anatase (101) surface. *Phys. Rev. Lett.* **109**, 136103 (2012).

14 Jang, W. L. *et al.* Growth and electronic properties of sputtered Ni–NiO films as a potential resistive memory. *Sci. Adv. Mater.* **5**, 1346–1353 (2013).
